# Healthcare professionals’ perspectives on medication adherence-supporting tools: a cross‑sectional survey in Italy

**DOI:** 10.1007/s00228-026-04074-y

**Published:** 2026-05-14

**Authors:** Monia Donati, Carlotta Lunghi, Giulia Grillini, Marco Domenicali, Maria Lia Lunardelli, Veronica Pasini, Susy Milandri, Monica Mussoni, Fabio Pieraccini, Elisa Sangiorgi, Emanuel Raschi, Valentina Colonnello, Elisabetta Poluzzi

**Affiliations:** 1 Department of Medical and Surgical Sciences, Alma Mater Studiorum, University of Bologna, Bologna, Italy; 2 Department of Life Sciences, Health and Health Professions, Link Campus University, Rome, Italy; 3 Geriatric Acute Care, Orthogeriatric Unit & Center for Diagnosis of Cognitive Disorders and Dementia, IRCCS-AOUBO, Bologna, Italy; 4 General Practitioner, AUSL Romagna, Cesena District, Cesena, Italy; 5 Local Health Authority of Romagna, Forlì, Italy; 6 Pharmaceutical Care Department, Local Health Authority of Romagna, Forlì, Italy; 7 Drug and Medical Devices Area, General Directorate for Personal Care, Health, and Welfare of the Emilia Romagna Region, Bologna, Italy

**Keywords:** Medication adherence, Older adults, Questionnaire, Barriers, Digital tools, Healthcare professionals

## Abstract

**Purpose:**

Medication adherence is essential for treatment effectiveness, yet the uptake of adherence-supporting tools in routine care remains suboptimal. This study assessed Italian healthcare professionals’ knowledge, perceived utility, and willingness to recommend medication adherence tools, and explored perceived barriers to their adoption.

**Methods:**

A questionnaire targeting healthcare professionals working in inpatient or outpatient settings across Italy was developed and validated by a panel of seven experts. It was anonymously distributed online between October and December 2023 to physicians, pharmacists, and nurses. The survey included open-ended and Likert-scale questions investigating use, perceived usefulness, barriers, and future willingness to recommend specific medication adherence tools. Data were analyzed using descriptive statistics, and differences by professional category were evaluated using Chi-square or Fisher’s exact tests. Open-ended responses were analyzed through a multi-step conventional content analysis independently conducted by two researchers.

**Results:**

A total of 660 healthcare professionals participated, including pharmacists (35%), nurses (26%), general practitioners (22%), geriatricians or internists (8.6%), and other medical doctors (8.5%). Overall, knowledge and recommendation regarding medication adherence tools were limited, with awareness ranging from 40.0% to 94.7% depending on the tool. Traditional tools such as pillboxes and paper diaries were more frequently used (42.1% and 26.3%, respectively) and were perceived as useful (26.7% and 22.3%). In contrast, digital tools (e.g., mobile apps, SMS reminders, electronic pillboxes) were rarely recommended and often described as unfamiliar. Patient-related barriers emerged as the most commonly reported obstacles for both traditional and digital tools.

**Conclusions:**

Italian healthcare professionals show limited familiarity with and use of medication adherence tools, particularly digital solutions. Targeted training and system-level strategies are needed to increase awareness, address perceived barriers, and promote the integration of effective adherence-supporting tools into routine care.

**Supplementary Information:**

The online version contains supplementary material available at 10.1007/s00228-026-04074-y.

## Introduction

Medication adherence is essential to ensure therapeutic effectiveness. Yet, two decades after the original WHO alarm, patients undergoing long-term treatments still struggle to adhere to healthcare professionals’ recommendations regarding their pharmacological regimens [1]. Indeed, a recent systematic review estimates that roughly 50% of adults prescribed medications for their chronic conditions deviate from recommended regimens [2]. Non-adherence is associated with increased mortality and hospitalizations [3–5]. For instance, a 2025 meta-analysis reported that poor adherence in hypertensive adults increased all-cause mortality by about 30% and cardiovascular mortality by 60% [6]. Similarly, those with diabetes and/or hypertension who adhered well had 28% to 32% lower odds of emergency visits and hospital admissions compared to those with low adherence [7]. Good medication adherence can lead to cost savings in healthcare. Indeed, studies show that improved adherence, although potentially increasing medication costs, can significantly reduce other medical expenses, including lower healthcare service utilization, decreased hospitalization rates, and fewer emergency room visits [8–12]. Demographic trends amplify the problem. Polypharmacy [13], a recognized predictor of non-adherence [14], impacts a significant proportion of Europeans aged 65 and older, surpassing 50% in various countries [15].

Various tools to support medication adherence have been developed, spanning from traditional to digital methods. Traditional tools, such as compartmental pillboxes and patient diaries, can be useful in managing medication schedules [16–20]. Digital tools, including electronic pillboxes, mobile apps, SMS reminders, and interactive voice response (IVR) systems, can enhance adherence rates and are increasingly prominent in clinical practice and have demonstrated effectiveness in various conditions, including hypertension and cardiovascular diseases [21–33]. Collectively, the literature demonstrates that multiple tool classes can improve adherence, with the effect size modulated by usability, behavioural content, and integration into existing clinical workflows [34, 35].

Despite evidence on their utility, the application of adherence technologies remains modest. In an ENABLE Pan-European survey of almost 3,000 clinicians, only 10–15% used digital solutions, while more than 90% still relied on direct patient counselling [36]. An umbrella review of systematic reviews confirmed that infrastructure deficits, insufficient training, and fears of additional workload predominate among barriers to the use of digital health technologies among healthcare professionals, even though the review did not focus on medication adherence tools [37].

To the best of our knowledge, no study has systematically explored the knowledge, utilization, and perceived barriers related to analogue or digital adherence tools among Italian healthcare professionals, including pharmacists, nurses, general practitioners, and specialist physicians. This knowledge gap is crucial given Italy’s recent digital health transition and its ageing, poly-medicated population, leading to an urgent need for effective adherence interventions. Generating profession-stratified, context-specific data will inform targeted training programs, guide technology implementation, and support policy initiatives aimed at incorporating adherence tools into routine clinical care. This study aimed to explore the knowledge, utilization, perceived utility, and future willingness/intention to use of tools developed to enhance patient medication adherence among healthcare professionals in Italy. It also sought to investigate perceived barriers to their implementation in clinical practice and potential differences among healthcare professions, as well as between traditional and digital tools.

## Materials and methods

### Questionnaire creation

We designed an anonymous online questionnaire targeting healthcare professionals employed in either inpatient or outpatient settings throughout Italy. Detailed information about the creation and content of the questionnaire has been previously published [38], and the questionnaire is available in the Supplementary material. Briefly, the questionnaire was divided into three sections. The first one gathered demographic and job-related information. The second section examined participants’ views on tools to improve patients’ adherence to medications, while the third section focused on tools associated with the appropriateness of prescribing. This study presents the results of the analyses from section two on medication adherence. Results from the other parts have been published elsewhere [38].

The list of tools included in section two was derived from a comprehensive literature review conducted via PubMed. This review aimed to identify the most frequently used tools for enhancing medication adherence (Supplementary Table S1). A panel of experts, comprising three pharmacists, one nurse, one general practitioner (GP), one internist, and one geriatrician, then validated the list to confirm its relevance within the Italian healthcare context. They also reviewed the questionnaire to ensure its completeness and excluded tools intended for specific purpose settings, opting instead for those more widely applicable to routine clinical practice in Italy. The final list comprised both traditional and digital tools to enhance medication adherence. Participants were asked to reflect on their knowledge and recommendations regarding the use of medication adherence tools over the previous 12 months. These questions employed 4-point or 5-point Likert scales (ranging from 1 – e.g., “never used” to 4 or 5 – e.g., “used for all patients”) with an additional option for “unknown”. Open-ended questions were designed to collect data on barriers related to the use of tools to improve medication adherence.

The finalized questionnaire was shared online via Microsoft Form^®^ from October to December 2023, and participants were recruited through a snowball sampling technique, beginning with healthcare professionals involved in the evaluation of the questionnaire.

### Statistical analysis

We conducted descriptive statistics to summarize categorical variables using absolute and percentage frequencies, while continuous variables were summarized by the median and interquartile range (IQR: Q1-Q3). Either the Chi-square test or Fisher’s exact test (depending on the data distribution) was used to compare tool usage among various professional categories (p-values were simulated through 2000 iterations when expected frequencies were insufficient for precise p-value computation). The Bonferroni correction was applied to account for multiple comparisons.

To facilitate interpretation, Likert scale responses were collapsed. For perceived utility, the original four-point scale (“extremely useful,” “very useful,” “slightly useful,” “not useful at all”) was dichotomized, with “extremely useful” and “very useful” classified as positive responses. The willingness to use in the future, measured on a four-point scale (“definitely yes,” “probably yes,” “probably not,” “definitely not”), was similarly dichotomized, with “definitely yes” and “probably yes” treated as positive responses. In both analyses, all positive responses across tools were pooled to form the denominator, and the proportion attributable to each tool was calculated. This approach provided the relative distribution of tools among all instances in which respondents expressed a favorable opinion. Analyses were also stratified by professional group.

Using a multi-step conventional content analysis approach [39], open-ended responses about the perceived barriers of each tool were systematically analyzed using a multi-step thematic approach. Initially, two researchers (CL and MD) independently categorized and coded the responses, followed by a reconciliation process with a third reviewer (VC) to address any discrepancies. This process guaranteed that all themes from the data were thoroughly captured. The research team reviewed any vague or unclear responses to establish the most suitable categorization, achieving excellent interrater reliability (Cohen κ > 0.80). Responses were first classified into three broad categories: patient-related, healthcare professional-related and tool-related. Then they were subclassified into more specific fields (e.g., usability of the tool among the tool-related barriers). No qualitative data analysis software was used.

## Results

Of the 680 collected responses, 20 respondents did not provide consent for their data to be used in the study, resulting in 660 valid responses for analysis. The majority were women (67.1%) and were aged between 40 and 60 (54.4%), compared to 28.4% under 40 and 17.1% over 60. Responders were employed as pharmacists (34.7%), nurses (26.2%), GPs (21.5%), geriatricians or internists (8.6%), and other medical doctors (MDs) (8.5%).

The median years of professional experience varied significantly across professions (*p* < 0.001). Pharmacists and other MDs exhibited the highest median experience (18 years; IQR: 9–27), while general practitioners (GPs) reported the least (10 years; IQR: 2–28). The weekly patient count also differed based on profession (*p* < 0.001), with pharmacists providing care for the most patients (300 [IQR: 150–500], including 175 [IQR: 80–300] patients over 65) and geriatricians/internists and other MDs treating the least number of patients (30; IQR: 25–50 and 30; IQR: 20–50, respectively). Overall, more than half of the patients under the care of all health professionals were aged 65 and above. Detailed information about the responders has been published elsewhere [38].

## Knowledge and recommendations of medication adherence-supporting tools

**Fig. 1:**
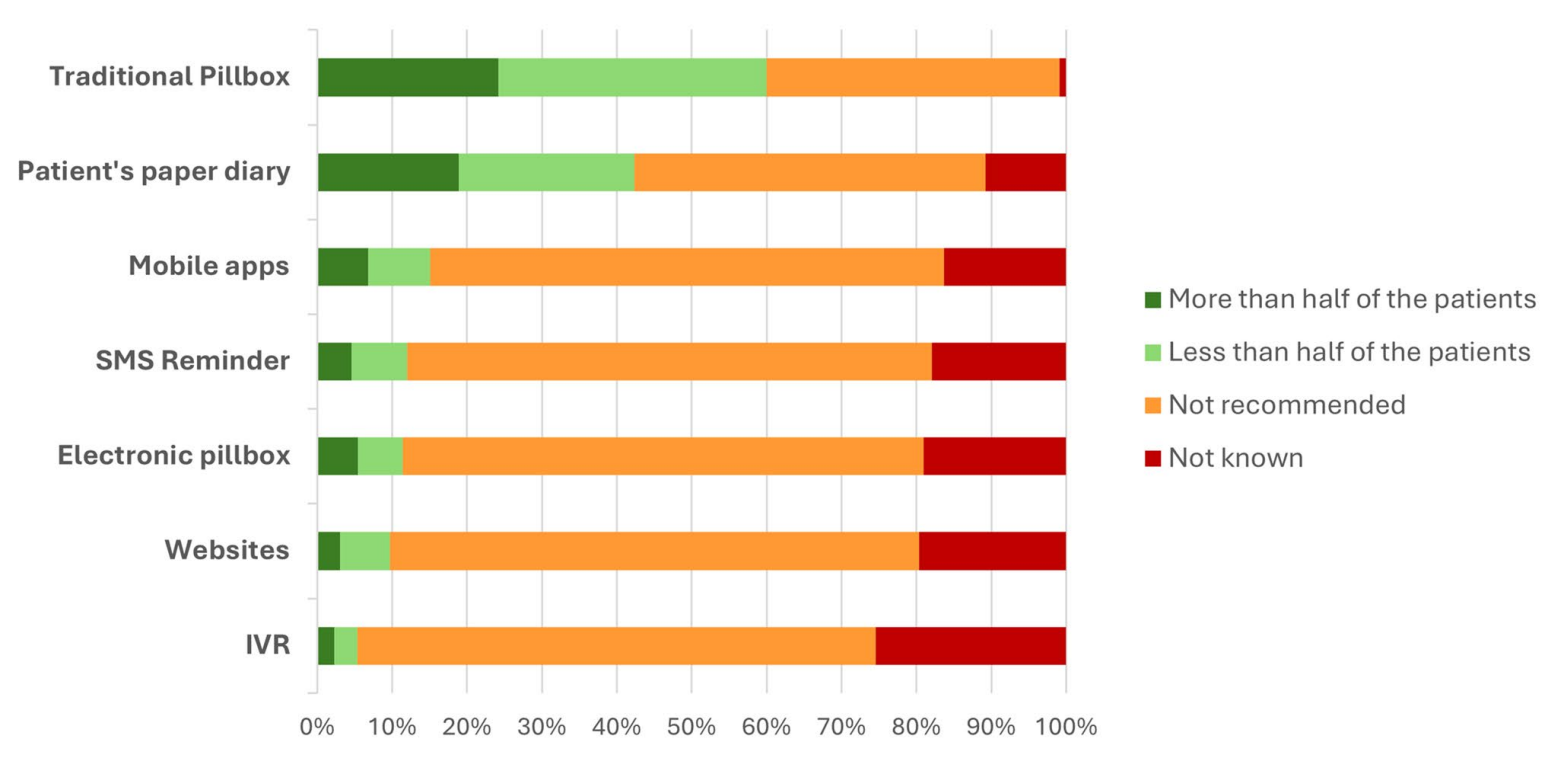
Knowledge and Recommendations of Medication Adherence Tools

**Fig. 2:**
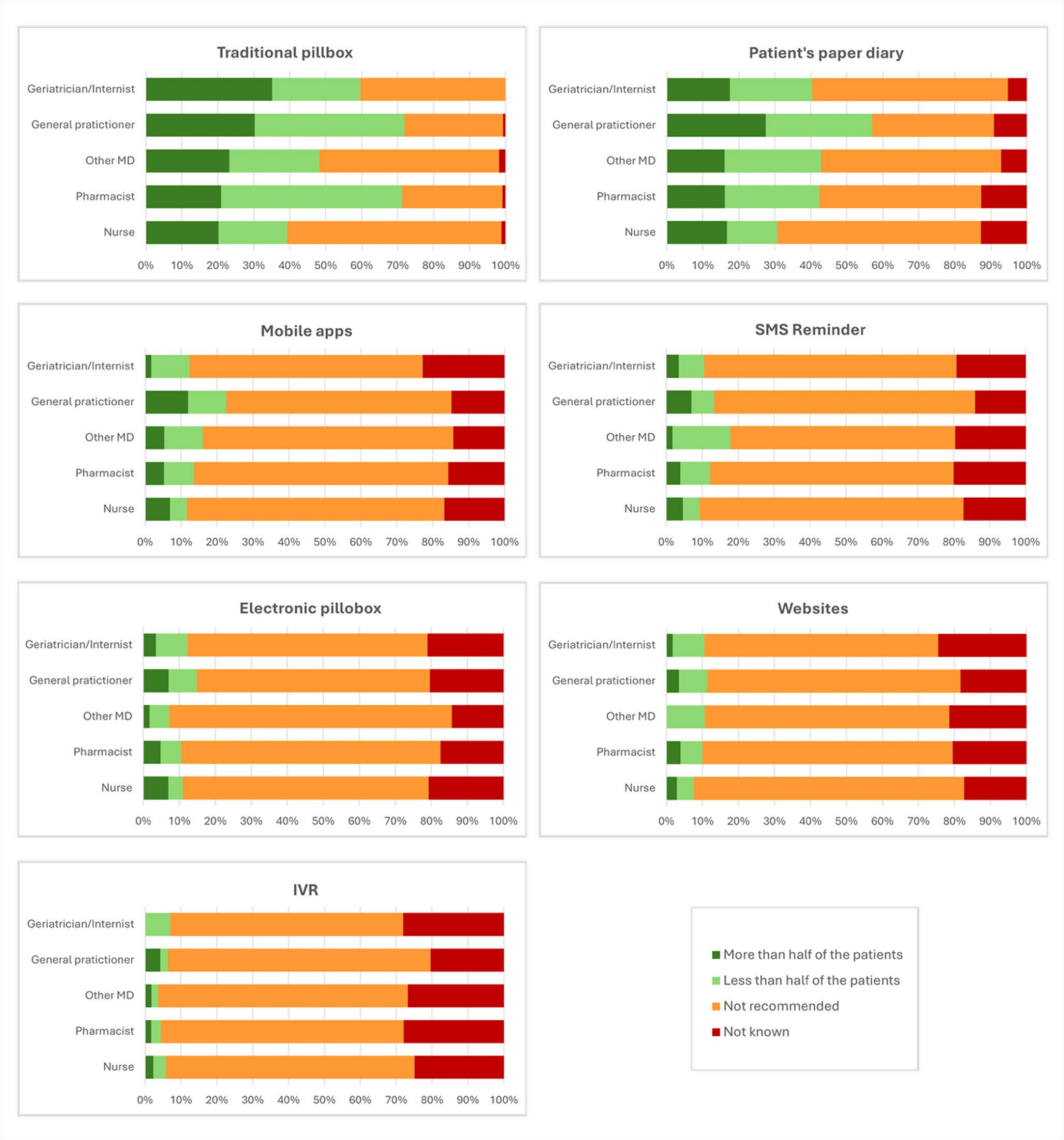
Knowledge and Recommendations of Medication Adherence-Supporting Tools by Profession

Knowledge and recommendations regarding tools to improve medication adherence were overall limited, ranging from 5.3% to 60.0% across the tools (Fig. 1; Table S2). “Not recommended” was the prevailing response for nearly all tools, with the main exception of the traditional pillbox by general practitioners and pharmacists (Fig. 2; Table S2). Traditional options (pillbox and paper diary) were relatively better known and more often suggested, though typically only for less than half of patients, irrespective of professional group. In contrast, digital solutions (mobile apps, SMS reminders, electronic pillboxes, websites, and IVR) were rarely recommended and often reported as “not known,” with only minor variations across specialties (Figs. 1 and 2; Tables S2–S3).

Information regarding knowledge and recommendations was collected using a 4-point Likert scale, ranging from 1 “never used” to 4 “used for all patients”, with an additional option for “unknown” if the respondent was not aware of the tool. Responses 3 (“more than half of the patients”) and 4 (“all patients”) were grouped together in a single category (“more than half of the patients”).

## Perceived utility and willingness to recommend medication adherence tools in the future

**Fig. 3:**
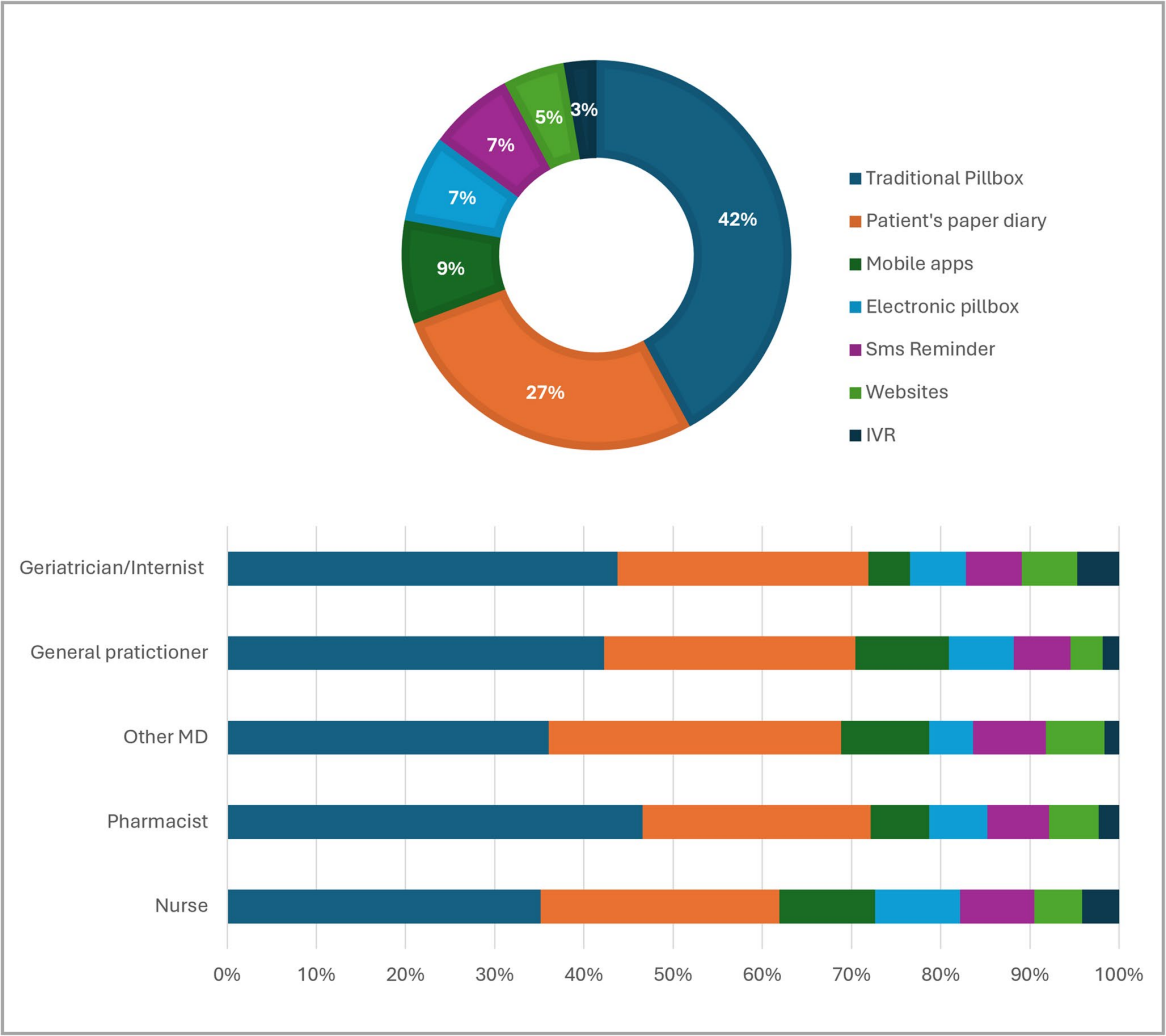
Perceived Utility to Use Medication Adherence-Supporting Tools

As specified in the Methods section, respondents were asked to rate the perceived utility of the tools they both knew and had recommended at least once, with “useful” defined as a positive response. The tool most frequently considered useful was the traditional pillbox (42.1%), followed by the patient’s diary (26.3%). Among digital options, mobile apps received the highest share of positive responses (8.6%), while electronic pillboxes, SMS reminders, websites, and IVR systems each accounted for less than 8%. Differences by profession were observed: general practitioners and geriatricians/internists predominantly valued traditional tools, pharmacists showed relatively greater recognition of digital options, and nurses attributed a higher proportion of positive responses to electronic pillboxes and SMS reminders (Fig. 3). More detailed information is reported in Supplemental Table S3.

**Fig. 4:**
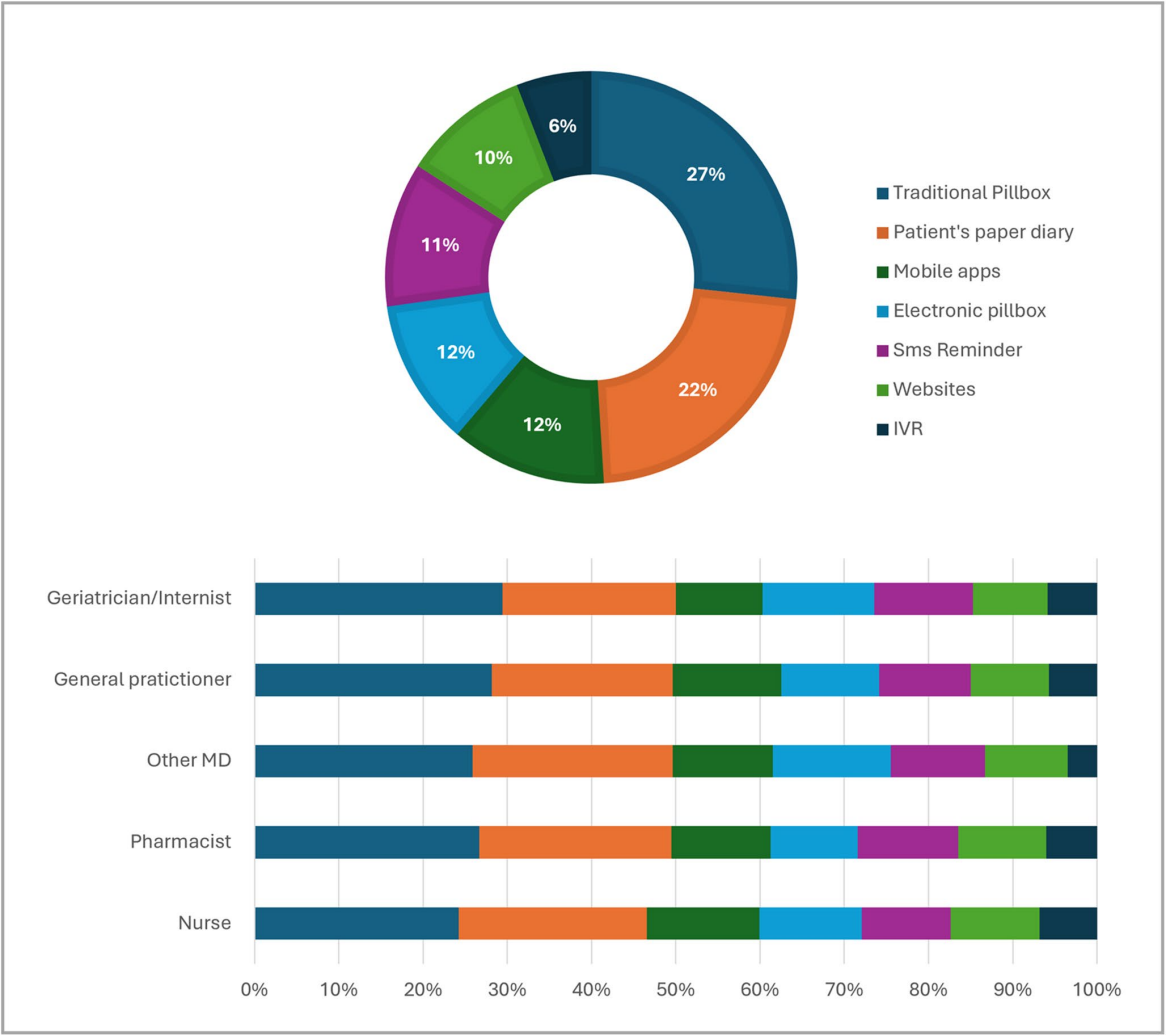
Willingness to Recommend Medication Adherence-Supporting Tools in the Future

Respondents were also asked about their willingness to recommend adherence tools in the future, considering only those tools they knew. The traditional pillbox (26.7%) and the patient’s paper diary (22.3%) received the largest shares of positive responses overall, followed by mobile apps (12.2%) and electronic pillboxes (11.5%). The distribution by profession was largely comparable, with only minor variations in the relative weight of digital tools (Fig. 4). Additional details are provided in Supplemental Table S4.

### Barriers to medication adherence-supporting tool adoption

**Fig. 5:**
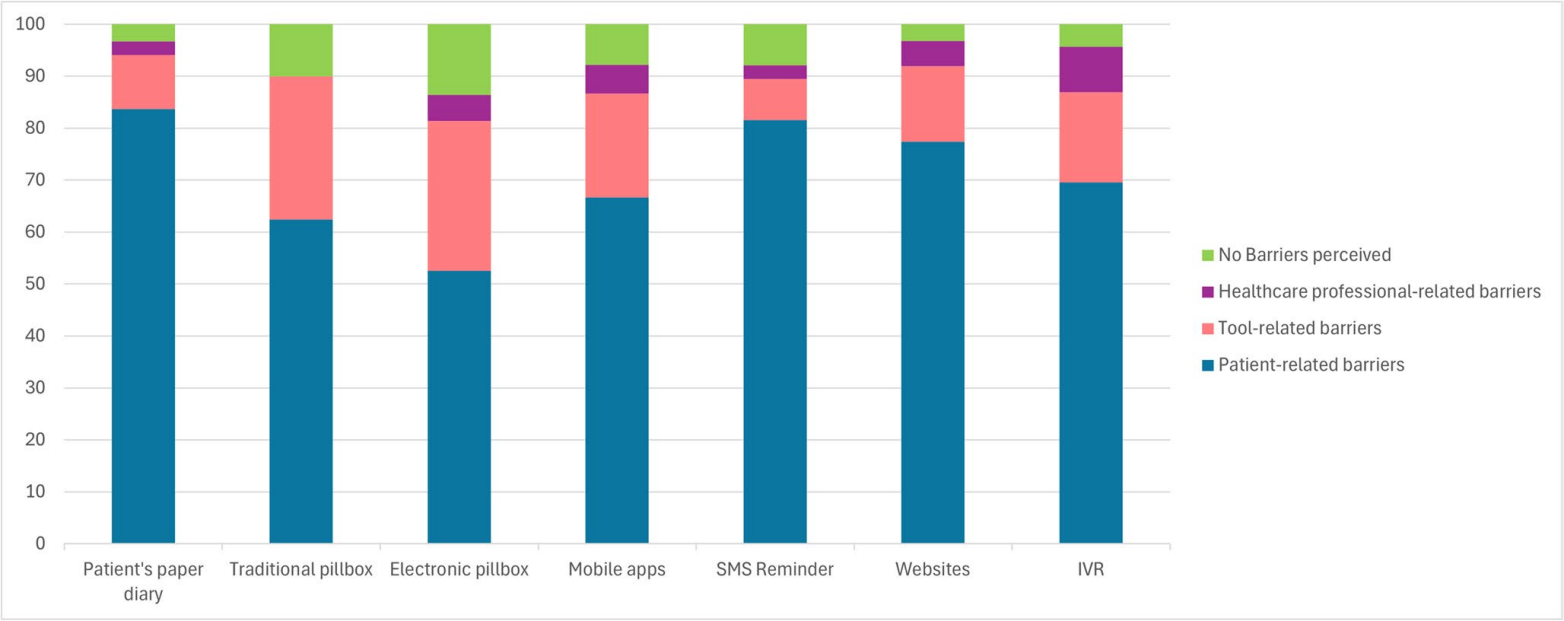
Distribution of perceived barriers to medication adherence-supporting tools across broad categories (patient-related, tool-related, and healthcare professional-related)

Participants also responded to open-ended questions about perceived barriers to using specific adherence-supporting tools. Among respondents who provided open-ended responses, most reported at least one barrier to using the adherence tools they recommended (Table 1). Patient-related barriers were the most frequently mentioned, particularly low motivation or adherence to the tool and age- or health-related limitations for traditional tools, and digital illiteracy for mobile apps, SMS reminders, websites, and IVR systems (Fig. 5; Table 1). Tool-related barriers, particularly poor usability, were reported for both traditional and digital tools, while cost issues emerged occasionally for electronic tools. Healthcare professional-related barriers, such as time constraints, limited knowledge of available tools, and suboptimal healthcare professional–patient relationship, were less common. Only a small minority of respondents indicated that they perceived no barriers at all, especially for pillboxes, both traditional and electronic.

**Table 1:** Perceived barriers to the use of medication adherence-supporting tools, by category and tool type, based on all reported responses

	Patient’s paper diary (Total = 337)	Traditional pillbox (Total = 410)	Electronic pillbox (Total = 59)	Mobile Apps (Total = 90)	SMS Reminder (Total = 76)	Websites (Total = 62)	IVR (Total = 23)
	*N* (%)	*N* (%)	*N* (%)	*N* (%)	*N* (%)	*N* (%)	*N* (%)
Patient-related barriers							
Poor tool adherence/motivation	116 (44.42)	55 (13.41)	3 (5.08)	5 (5.56)	7 (9.21)	7 (11.29)	2 (8.70)
Age- and health-related limitations	92 (27.30)	110 (26.83)	8 (13.56)	7 (7.78)	14 (18.42)	7 (11.29)	9 (39.13)
Socio-cultural constraints	43 (12.76)	4 (0.98)	0 (0)	1 (1.11)	1 (1.32)	3 (4.84)	1 (4.35)
Dependence on caregiver	31 (9.20)	87 (21.22)	2 (3.39)	4 (4.44)	0 (0)	3 (4.84)	1 (4.35)
Digital illiteracy	0 (0)	0 (0)	18 (30.51)	43 (47.78)	40 (52.63)	28 (45.16)	3 (13.04)
**Tool-related barriers**							
Poor tool usability	35 (10.39)	108 (26.34)	11 (18.64)	18 (20.00)	6 (7.89)	8 (12.90)	4 (17.39)
Tool-related costs	0 (0)	5 (1.22)	6 (10.17)	0 (0)	0 (0)	1 (1.61)	0 (0)
**Healthcare professional-related barriers**							
Time constraints	5 (1.48)	0 (0)	0 (0)	3 (3.33)	2 (2.63)	3 (4.84)	0 (0)
Poor tool knowledge	0 (0)	0 (0)	3 (5.08)	0 (0)	0 (0)	0 (0)	0 (0)
Suboptimal relationship with the patient	4 (1.19)	0 (0)	0 (0)	2 (2.22)	0 (0)	0 (0)	2 (8.70)
**No Barriers perceived**	11 (3.26)	41 (10.00)	8 (13.56)	7 (7.78)	6 (7.89)	2 (3.23)	1 (4.35)

Number of respondents: Patient’s paper diary = 241; Traditional pillbox = 329; Mobile Apps = 73; SMS Reminder = 66; Websites = 48; IVR = 23; N: number of respondents. Total: total number of responses

## Discussion

To our knowledge, this is the first study to investigate healthcare professionals’ perceptions, knowledge, and utilization of tools designed to improve medication adherence in the Italian context, offering insights that may guide future research and inform policy initiatives.

We found that, despite evidence supporting their effectiveness [34], most adherence tools were poorly known by healthcare professionals and rarely recommended in the Italian context. Pillboxes and patient diaries were relatively well known and more frequently used, though typically used for fewer than half of patients. Their simplicity, low cost, and ease of integration into daily routines likely explain this result. Mobile apps, SMS reminders, electronic pillboxes, websites, and IVR systems were rarely recommended and were often unknown to healthcare professionals. This reflects both limited dissemination and a lack of familiarity or confidence in digital health solutions. Evidence on clinician engagement with digital adherence tools is limited, but what exists highlights similar barriers to those suggested by our results. For instance, qualitative work in primary care settings shows that healthcare professionals often experience time constraints, competing clinical demands, and infrastructural limitations that hinder systematic attention to non-adherence, even when risk is recognized [40]. Furthermore, awareness and recommendation of digital health apps among pharmacists vary, with substantial proportions acknowledging their usefulness yet not routinely recommending them, often due to limited knowledge or confidence in these tools [41].

This gap between research evidence and clinical practice may be addressed by investing in healthcare professional education, not only at the academic level but also through continuous professional development and training, and possibly through interdisciplinary approaches. Evidence from a large systematic review of healthcare professionals’ adoption of digital health technologies indicates that training/educational programmes are consistently associated with improved uptake. In contrast, infrastructure barriers and workload-related concerns are among the most frequently reported barriers [37]. While minor differences emerged in our study (e.g., pharmacists and nurses showing slightly more openness to digital options), the overall pattern of low knowledge and limited utilization was consistent across professional groups. This suggests that barriers are systemic rather than profession-specific.

Consistent with the observed results, healthcare professionals in our sample also rated traditional adherence tools higher in perceived utility and future willingness to use than digital tools. This preference for familiar, low‑tech strategies likely reflects pragmatic considerations in clinical practice, where tools that can be implemented immediately, without additional training or workflow disruption, are more readily adopted. Across disciplines, the relatively low endorsement of digital tools suggests that professionals may perceive such innovations as less feasible or less integrated into routine care. This pattern aligns with broader evidence indicating that many digital interventions for medication adherence show inconsistent adoption in real‑world settings [42]. While digital interventions can improve adherence outcomes in some settings, their effectiveness is often mixed and depends on user engagement, design quality, and integration into care workflows rather than on the technology itself. These challenges include accessibility, acceptability, and the need for structured support frameworks to prevent the exacerbation of inequities in healthcare delivery [42].

In our sample, some healthcare professionals noted the difficulty of recommending the use of digital tools to older patients, who often have not only low digital literacy but also limited education. Nevertheless, some evidence suggests that tools specifically developed for older patients exist and can improve patient adherence and clinical outcomes [43]. These concerns are consistent with broader population-level indicators of digital competence in Italy. In the latest European Commission Digital Decade Country Report for Italy, basic digital skills remain below the EU average, with only 54.3% of Italian citizens having basic or above basic digital skills [44]. This indicates that limited digital competence is a structural constraint rather than an isolated clinical barrier. In this context, recommending app-, web-, SMS-, or IVR-based adherence tools to older and multimorbid patients is likely to require additional facilitation (training, caregiver involvement, and simplified onboarding) to avoid selective uptake and the risk of widening inequities.

The Italian healthcare setting may further amplify these structural barriers. High clinical workloads and limited protected time for preventive and supportive interventions such as adherence counselling characterize Italy’s primary care system. This may reduce clinicians’ opportunities to explore, introduce, and follow up on adherence tools, particularly digital ones that require initial setup and explanation. In addition, variation in digital health capacity across regions and within practice settings can contribute to unequal access to technological supports, further discouraging routine use of digital adherence solutions.

## Strengths and limitations

This study involved a wide range of professional groups working in diverse care settings, and we believe it provided an overview that reflects different perspectives and practices. The mixed design, combining closed questions with open-ended responses, enabled the analysis to move beyond descriptive statistics and highlight underlying attitudes and perceived barriers.

Nevertheless, some limitations must be considered. Because the survey relied on self-reporting, inaccuracies linked to memory or a tendency to provide socially desirable answers cannot be excluded. However, the consistently low awareness and utilization of adherence tools across professions suggest that these biases are unlikely to have substantially changed the overall picture. The findings should also be interpreted within the Italian context, as differences in healthcare organization, professional responsibilities, and cultural views of medication use may limit generalizability to other systems. In addition, the snowball recruitment strategy, with respondents’ anonymity by design, did not permit calculation of a response rate and may have affected representativeness. Lastly, the questionnaire was not subjected to formal validation procedures. Nevertheless, it was extensively revised with expert input to ensure relevance for everyday clinical practice in Italy.

## Conclusions

Medication adherence is a key factor in the effective treatment of chronic conditions, especially in older adults. Our study shows that medication adherence-supporting tools are poorly adopted and recommended by healthcare professionals in the Italian clinical settings. Low penetration of these tools into daily clinical practice exists. Bridging this gap requires targeted training programs, better dissemination of available tools, and integration into routine workflows. Without structural support, digital innovations may risk remaining underused despite their potential.

## Supplementary Information

Below is the link to the electronic supplementary material.

Supplementary Material 1 (DOCX 432 KB)

## Data Availability

Questionnaire data are anonymized at the source and are available upon written request to the corresponding author.
