# Supplementary material for: Healthcare professionals’ perspectives on medication adherence-supporting tools: a cross‑sectional survey in Italy
Source: Eur J Clin Pharmacol. 2026 May 14;82(6):151. doi: 10.1007/s00228-026-04074-y (PMC13171734; doi:10.1007/s00228-026-04074-y)
Supplement: Supplementary file 1 — Supplementary Material 1 (DOCX 432 KB) [file 228_2026_4074_MOESM1_ESM.docx]

**Supplementary material of the article:**

**Healthcare Professionals’ Perspectives on Medication Adherence-Supporting Tools: A Cross‑Sectional Survey in Italy**

Monia Donati, Carlotta Lunghi, Giulia Grillini, Marco Domenicali, Maria Lia Lunardelli, Veronica Pasini, Susy Milandri, Monica Mussoni, Fabio Pieraccini, Elisa Sangiorgi, Emanuel Raschi, Valentina Colonnello, Elisabetta Poluzzi

**Table S1** **– Articles retrieved in the literature review, along with the reported medication adherence-supporting tools**

| **Type of tools** | **Article** | **Author (first)** | **Year** |
| --- | --- | --- | --- |
| Drug packaging systems | "Electronic medication packaging devices and medication  adherence: A systematic review" | Kyle D. Checchi | 2014 |
| Interactive Voice Response (IVR) tools | "Interactive voice response interventions  targeting behaviour change: a systematic  literature review with meta-analysis  and meta-regression" | Stergiani Tsoli | 2018 |
| Educational interventions, remote monitoring, paper diary | "Effects of eHealth Interventions on Medication Adherence: A Systematic Review of the Literature" | Annemiek J Linn | 2011 |
| Remote monitoring | "Mobile Text Messaging for Health: A Systematic Review of  Reviews" | Amanda K. Hall | 2015 |
| Mobile apps | "Effectiveness of Mobile Medical Apps in Ensuring Medication Safety Among Patients With Chronic Diseases:Systematic Review and Meta-analysis" | Ting ting Zhou | 2022 |
| Remote monitoring | "The effect of electronic monitoring feedback on medication adherence and clinical outcomes: A systematic review" | Milou van Heuckelum | 2017 |
| Paper reminders | "Manually-generated reminders delivered on paper: eects on professional practice and patient outcomes" | Tomas Pantoja | 2019 |
| Electronic reminders (audiovisual) | "The effectiveness of interventions using electronic reminders to improve adherence to chronic medication: a systematic review of the literature" | Marcia Vervloet | 2012 |
| Educational interventions, remote monitoring | "A systematic scoping review of interventions to optimise medication prescribing and adherence in older adults with cancer" | Melanie Murphy | 2022 |
| Educational interventions | "Patient-centered Outcomes of Medication Adherence  Interventions: Systematic Review and Meta-Analysis" | Vicki S. Conn | 2016 |
| Remote monitoring | "Modes of delivery for interventions to improve cardiovascular medication adherence: Review" | Sarah L. Cutrona | 2010 |
| Remote monitoring | "Helping patients help themselves: A systematic review of self-management support strategies in primary health care practice" | Sarah Dineen-Griffin | 2019 |
| Educational interventions | "Interventions for improving outcomes in patients with multimorbidity in primary care and community settings (Review)" | Smith SM | 2021 |
| Educational interventions, remote monitoring, electronic reminders (audiovisual) | "Technology-mediated interventions for enhancing medication adherence" | Niraj Mistry | 2015 |
| Educational interventions | "Interventions to improve safe and elective medicines use by consumers: an overview of systematic reviews (Review)" | Ryan RE | 2022 |
| Educational interventions, paper reminders, electronic reminders (audiovisual) | "Interventions to improve adherence to anti-osteoporosis medications: an updated systematic review" | D. Cornelissen | 2020 |
| Educational interventions | "Interventions for improving medication-taking ability and adherence in older adults prescribed multiple medications (Review)" | Cross AJ | 2020 |
| Remote monitoring | "Intervention Strategies for Improving Patient Adherence to Follow-Up in the Era of Mobile Information Technology: A Systematic Review and Meta-Analysis" | Haotian Lin | 2014 |
| Educational interventions, electronic reminders (audiovisual) | "A systematic overview of systematic reviews evaluating  medication adherence interventions" | Laura J. Anderson | 2020 |
| Electronic reminders (pillbox), drug packaging systems | "Packaging interventions to increase medication adherence:  systematic review and meta-analysis" | Vicki S. Conn | 2016 |
| Educational interventions, electronic reminders (pillbox) | "Health care provider targeted interventions to improve  medication adherence: systematic review and meta-analysis" | Vicki S. Conn | 2017 |
| Educational interventions, remote monitoring, drug packaging systems | "Pharmacist-led Interventions to Improve Medication Adherence  in Older Adults: A Meta-Analysis" | Zachary A. Marcum | 2022 |
| Educational intervenions, electronic devices (pillbox, blood glucose concentration monitoring systems) | "Pharmacy-led interventions to improve medication adherence among adults with diabetes: A systematic review and meta-analysis" | Bobby Presley | 2019 |
| Mobile apps (ALICE, MediSAFE, MyIDEA education  app, MedApp-CHD, MyPso Smartop) | "Do mobile device apps designed to  support medication adherence  demonstrate efficacy? A systematic  review of randomised controlled trials,  with meta-analysis" | Laura Catherine Armitage | 2020 |
| Mobile apps (MediSAFE, MyTherapy) | "Aplicaciones móviles para mejorar la adherencia a la medicación: revisión y análisis de calidad" | Beatriz González de León | 2021 |
| Interactive Voice Reponse (IVR) tools | "Positive influence of short message service and voice call interventions on adherence and health outcomes in case of chronic disease care: a systematic review" | F. Yasmin | 2016 |
| Educational interventions | "Theory-Based Interventions to Improve Medication Adherence in Older Adults Prescribed Polypharmacy: A Systematic Review" | Deborah E. Patton | 2017 |
| Educational interventions, pillbox, paper diary | "Medication Adherence Interventions That Target Subjects with  Adherence Problems: Systematic Review and Meta-analysis" | Vicki S. Conn | 2015 |
| Educational interventions, electronic reminders (pillbox) | "Nurse interventions to improve medication adherence among discharged older adults: a systematic review" | HENK VERLOO | 2017 |
| Educational interventions (websites) remote monitoring, pillbox, paper diary, audiovisual devices for reminder | "Interventions for enhancing medication adherence (Review)" | Nieuwlaat R | 2014 |
| Remote monitoring | "Intervention Strategies for Improving Patient Adherence to Follow-Up in the Era of Mobile Information Technology: A Systematic Review and Meta-Analysis" | Haotian Lin | 2014 |
| Educational interventions, remote monitoring, audiovisual devices for reminder, pillobox, e-pillbox, drug packaging systems | "A systematic overview of systematic reviews evaluating  medication adherence interventions" | Laura J. Anderson | 2020 |
| Educational interventions | "Interventions for improving medication-taking ability and adherence in older adults prescribed multiple medications (Review)" | Cross AJ | 2020 |
| Educational interventions | "Interventions to improve adherence to anti-osteoporosis medications: an updated systematic review" | D. Cornelissen | 2020 |
| Educational interventions, remote monitoring | "Interventions to improve safe and e ective medicines use by consumers: an overview of systematic reviews (Review)" | Ryan RE | 2022 |
| Educational interventions | "Interventions for improving outcomes in patients with multimorbidity in primary care and community settings (Review)" | Smith SM | 2021 |
| Educational interventions, remote monitoring (SMS) | "Helping patients help themselves: A systematic review of self-management support strategies in primary health care practice" | Sarah Dineen-Griffin | 2019 |
| Remote monitoring (calls and SMS), electronic reminders (pillbox), home blood pressure monitors (HBPM), e-health | "Technology-mediated interventions for enhancing medication adherence" | Niraj Mistry | 2015 |
| Educational interventions, remote monitoring (calls and SMS), paper remindrs, electronic reminders | "Modes of delivery for interventions to improve cardiovascular medication adherence: Review" | Sarah L. Cutrona | 2011 |
| Educational interventions (websites, gamification), remote monitoring, paper diary, electronic reminders | "What are the important design features  of personal health records to improve  medication adherence for patients with  long-term conditions? A systematic  literature review" | Elisavet Andrikopoulou | 2018 |
| Educational interventions (websites), remote monitoring | "Nurses' responsibilities and tasks in pharmaceutical care:  A scoping review" | Elyne De Baetselier | 2018 |
| Educational interventions | "Motivational Interviewing Improves Medication Adherence: a Systematic Review and Meta-analysis" | Ana Palacio | 2016 |
| Educational interventions | "Is patient empowerment the key to promote adherence? A systematic review of the relationship between self-efficacy, health locus of control and medication adherence" | Lilla Na´fra´di | 2017 |
| Mobile apps (education and reminder) | "Medication management support in diabetes: a systematic assessment of diabetes self-management apps" | Zhilian Huang1 | 2019 |
| Educational interventions, remote monitoring, reminders | "Interventions for improving adherence to treatment recommendations in people with type 2 diabetes mellitus (Review)" | Vermeire EIJJ | 2009 |
| Educational interventions, remote monitoring, reminders | "Impact of DSMES app interventions  on medication adherence in type 2  diabetes mellitus: systematic review  and meta-analysis" | Dumisani Enricho Nkhoma | 2021 |
| Educational intrventions, remote monitoring | "Interventions to improve adherence to lipid-lowering medication (Review)" | van Driel ML | 2016 |
| Remote monitoring (SMS, calls, IVR) | "Mobile phone-based interventions for improving adherence to medication prescribed for the primary prevention of cardiovascular disease in adults (Review)" | Palmer MJ | 2021 |
| Educational interventions, remote monitoring | "Effect of pragmatic versus explanatory  interventions on medication adherence  in people with cardiometabolic  conditions: a systematic review  and meta-analysis" | Claire Fitzpatrick | 2020 |
| Educational interventions, remote monitoring, reminders, drug packaging systems | "Cardiovascular medication: improving adherence" | Liam Glynn and Tom Fahey | 2010 |
| Educational interventions | "Pharmacists and medication adherence in asthma: a systematic review and meta-analysis" | Marissa Ayano Mes | 2018 |
| Educational interventons, remote monitoring, paper reminders, electronic reminders | "Interventions to improve medication adherence in tuberculosis patients: a systematic review of randomized controlled studies" | Ivan S. Pradipta | 2020 |
| Educational interventions, reminders | "Improving medication adherence in chronic obstructive pulmonary disease: a systematic review" | Jamie Bryant | 2013 |
| Educational interventions, remote monitoring, IVR, electronic reminders (SMS, pillbox, audiovisual), mobile apps | "The role of mHealth for improving medication adherence in patients with cardiovascular disease: a systematic review" | Yousuf Gandapur | 2016 |
| Educational interventions (websites), remote monitoring, IVR, electronic reminders (audiovisual, SMS), mobile apps | "Impact of eHealth on medication adherence among patients with asthma: A systematic review and meta-analysis" | Ruth Jeminiwa | 2019 |
| Educational interventions, remote monitoring, paper diary, reminders (SMS, calls) | "Effective components of self-management programs for chronic  obstructive pulmonary disease patients: scoping review" | Rita Georges Nohra | 2020 |
| Educational interventions, remote monitoring | "Pharmacists’ Role in Older Adults’ Medication Regimen Complexity: A Systematic Review" | Catharine Falch | 2021 |
| Educational interventions, remote monitoring, electronic reminders (SMS) | "Strategies for improving adherence to antiepileptic drug treatment in people with epilepsy (Review)" | Al-aqeel | 2020 |
| IVR, remote monitoring | "A systematic review and meta-analysis in the effectiveness of mobile phone interventions used to improve adherence to antiretroviral therapy in HIV infection" | Reshma Shah | 2019 |
| Educational interventions, remote monitoring, electronic reminders (SMS, pillbox, audiovisual) | "Interventions to Improve Adherence to Antiretroviral Therapy (ART) in Sub-Saharan Africa: An Updated Systematic Review" | Panmial Priscilla Damulak | 2021 |
| IVR, electronic reminders (SMS, calls) | "Efectiveness of mobile text reminder  in improving adherence to medication, physical  exercise, and quality of life in patients living  with HIV: a systematic review" | Sam Chidi Ibeneme | 2021 |

**Table S2** – **Knowledge and Recommendation of Medication Adherence-Supporting Tools**.

| How many patients over 65 have you recommended in the last months to use the following tools? | ALL  N=657 | Geriatrician/Internist  N = 57 | General pratictioner  N = 142 | Other MD  N = 56 | Pharmacist  N = 229 | Nurse  N = 173 | p-value | q-value* |
| --- | --- | --- | --- | --- | --- | --- | --- | --- |
| Patient's paper diary |  |  |  |  |  |  | 0.001 | 0.010 |
| **More than half of the patients** | 124 (18.87%) | 10 (17.54%) | 39 (27.46%) | 9 (16.07%) | 37 (16.16%) | 29 (16.76%) |  |  |
| **Less than half of the patients** | 154 (23.44%) | 13 (22.81%) | 42 (29.58%) | 15 (26.79%) | 60 (26.20%) | 24 (13.87%) |  |  |
| **Not recommended** | 308 (46.88%) | 31 (54.39%) | 48 (33.80%) | 28 (50.00%) | 103 (44.98%) | 98 (56.65%) |  |  |
| **Not known** | 71 (10.81%) | 3 (5.26%) | 13 (9.15%) | 4 (7.14%) | 29 (12.66%) | 22 (12.72%) |  |  |
| Traditional Pillbox |  |  |  |  |  |  | <0.001 | 0.003 |
| **More than half of the patients** | 159 (24.20%) | 20 (35.09%) | 43 (30.28%) | 13 (23.21%) | 48 (20.96%) | 35 (20.23%) |  |  |
| **Less than half of the patients** | 235 (35.77%) | 14 (24.56%) | 59 (41.55%) | 14 (25.00%) | 115 (50.22%) | 33 (19.08%) |  |  |
| **Not recommended** | 257 (39.12%) | 23 (40.35%) | 39 (27.46%) | 28 (50.00%) | 64 (27.95%) | 103 (59.54%) |  |  |
| **Not known** | 6 (0.91%) | 0 (0.00%) | 1 (0.70%) | 1 (1.79%) | 2 (0.87%) | 2 (1.16%) |  |  |
| Electronic pillbox |  |  |  |  |  |  | 0.800 | 1.000 |
| **More than half of the patients** | 36 (5.48%) | 2 (3.51%) | 10 (7.04%) | 1 (1.79%) | 11 (4.80%) | 12 (6.94%) |  |  |
| **Less than half of the patients** | 39 (5.94%) | 5 (8.77%) | 11 (7.75%) | 3 (5.36%) | 13 (5.68%) | 7 (4.05%) |  |  |
| **Not recommended** | 457 (69.56%) | 38 (66.67%) | 92 (64.79%) | 44 (78.57%) | 165 (72.05%) | 118 (68.21%) |  |  |
| **Not known** | 125 (19.03%) | 12 (21.05%) | 29 (20.42%) | 8 (14.29%) | 40 (17.47%) | 36 (20.81%) |  |  |
| SMS Reminder |  |  |  |  |  |  | 0.500 | 1.000 |
| **More than half of the patients** | 30 (4.57%) | 2 (3.51%) | 10 (7.04%) | 1 (1.79%) | 9 (3.93%) | 8 (4.62%) |  |  |
| **Less than half of the patients** | 49 (7.46%) | 4 (7.02%) | 9 (6.34%) | 9 (16.07%) | 19 (8.30%) | 8 (4.62%) |  |  |
| **Not recommended** | 460 (70.02%) | 40 (70.18%) | 103 (72.54%) | 35 (62.50%) | 155 (67.69%) | 127 (73.41%) |  |  |
| **Not known** | 118 (17.96%) | 11 (19.30%) | 20 (14.08%) | 11 (19.64%) | 46 (20.09%) | 30 (17.34%) |  |  |
| IVR |  |  |  |  |  |  | 0.800 | 1.000 |
| **More than half of the patients** | 15 (2.28%) | 0 (0.00%) | 6 (4.23%) | 1 (1.79%) | 4 (1.75%) | 4 (2.31%) |  |  |
| **Less than half of the patients** | 20 (3.04%) | 4 (7.02%) | 3 (2.11%) | 1 (1.79%) | 6 (2.62%) | 6 (3.47%) |  |  |
| **Not recommended** | 455 (69.25%) | 37 (64.91%) | 104 (73.24%) | 39 (69.64%) | 155 (67.69%) | 120 (69.36%) |  |  |
| **Not known** | 167 (25.42%) | 16 (28.07%) | 29 (20.42%) | 15 (26.79%) | 64 (27.95%) | 43 (24.86%) |  |  |
| Websites |  |  |  |  |  |  | 0.500 | 1.000 |
| **More than half of the patients** | 20 (3.04%) | 1 (1.75%) | 5 (3.52%) | 0 (0.00%) | 9 (3.93%) | 5 (2.89%) |  |  |
| **Less than half of the patients** | 44 (6.70%) | 5 (8.77%) | 11 (7.75%) | 6 (10.71%) | 14 (6.11%) | 8 (4.62%) |  |  |
| **Not recommended** | 464 (70.62%) | 37 (64.91%) | 100 (70.42%) | 38 (67.86%) | 159 (69.43%) | 130 (75.14%) |  |  |
| **Not known** | 129 (19.63%) | 14 (24.56%) | 26 (18.31%) | 12 (21.43%) | 47 (20.52%) | 30 (17.34%) |  |  |
| Mobile apps |  |  |  |  |  |  | 0.200 | 1.000 |
| **More than half of the patients** | 45 (6.85%) | 1 (1.75%) | 17 (11.97%) | 3 (5.36%) | 12 (5.24%) | 12 (6.94%) |  |  |
| **Less than half of the patients** | 54 (8.22%) | 6 (10.53%) | 15 (10.56%) | 6 (10.71%) | 19 (8.30%) | 8 (4.62%) |  |  |
| **Not recommended** | 451 (68.65%) | 37 (64.91%) | 89 (62.68%) | 39 (69.64%) | 162 (70.74%) | 124 (71.68%) |  |  |
| **Not known** | 107 (16.29%) | 13 (22.81%) | 21 (14.79%) | 8 (14.29%) | 36 (15.72%) | 29 (16.76%) |  |  |

*Q-value stands for the p-value corrected for multiple testing through the Bonferroni correction. MD: medical doctor.

**Table S3** – **Perceived Utility to Use Medication Adherence-Supporting Tools**

| How useful do you consider the use of the following tools in support treatment  Adherence? | ALL  N=657 | Geriatrician/Internist  N = 57 | General pratictioner  N = 142 | Other MD  N = 56 | Pharmacist  N = 229 | Nurse  N = 173 | p-value | q-value |
| --- | --- | --- | --- | --- | --- | --- | --- | --- |
| Patient's paper diary |  |  |  |  |  |  | 0.70 | 1.00 |
| **Extremely useful** | 38 (5.78%) | 2 (3.51%) | 13 (9.15%) | 1 (1.79%) | 15 (6.55%) | 7 (4.05%) |  |  |
| **Very useful** | 185 (28.16%) | 16 (28.07%) | 49 (34.51%) | 19 (33.93%) | 63 (27.51%) | 38 (21.97%) |  |  |
| **Slightly useful** | 51 (7.76%) | 5 (8.77%) | 16 (11.27%) | 4 (7.14%) | 19 (8.30%) | 7 (4.05%) |  |  |
| **Not useful at all** | 4 (0.61%) | 0 (0.00%) | 3 (2.11%) | 0 (0.00%) | 0 (0.00%) | 1 (0.58%) |  |  |
| **N/A** | 379 (57.69%) | 34 (59.65%) | 61 (42.96%) | 32 (52.14%) | 132 (57.64%) | 120 (69.36%) |  |  |
| Traditional Pillbox |  |  |  |  |  |  | 0.80 | 1.00 |
| **Extremely useful** | 74 (11.26%) | 6 (10.53%) | 22 (15.49%) | 4 (7.14%) | 27 (11.79%) | 15 (8.67%) |  |  |
| **Very useful** | 270 (41.10%) | 22 (38.60%) | 71 (50.00%) | 18 (32.14%) | 115 (50.22%) | 44 (25.43%) |  |  |
| **Slightly useful** | 50 (7.61%) | 6 (10.53%) | 9 (6.34%) | 5 (8.93%) | 21 (9.17%) | 9 (5.20%) |  |  |
| **Not useful at all** | 0 (0.00%) | 0 (0.00%) | 0 (0.00%) | 0 (0.00%) | 0 (0.00%) | 0 (0.00%) |  |  |
| **N/A** | 263 (40.03%) | 23 (40.35%) | 40 (28.17%) | 29 (51.79%) | 66 (28.82%) | 105 (60.69%) |  |  |
| Electronic pillbox |  |  |  |  |  |  | >0.9 | 1.00 |
| **Extremely useful** | 16 (2.44%) | 1 (1.75%) | 5 (3.52%) | 1 (1.79%) | 5 (2.18%) | 4 (2.31%) |  |  |
| **Very useful** | 43 (6.54%) | 3 (5.26%) | 11 (7.75%) | 2 (3.57%) | 15 (6.55%) | 12 (6.94%) |  |  |
| **Slightly useful** | 13 (1.98%) | 3 (5.26%) | 3 (2.11%) | 1 (1.79%) | 3 (1.31%) | 3 (1.73%) |  |  |
| **Not useful at all** | 3 (0.46%) | 0 (0.00%) | 2 (1.41%) | 0 (0.00%) | 1 (0.44%) | 0 (0.00%) |  |  |
| **N/A** | 582 (88.58%) | 50 (87.72%) | 121 (85.21%) | 52 (92.86%) | 205 (89.52%) | 154 (89.02%) |  |  |
| SMS Reminder |  |  |  |  |  |  | 0.50 | 1.00 |
| **Extremely useful** | 9 (1.37%) | 0 (0.00%) | 3 (2.11%) | 0 (0.00%) | 5 (2.18%) | 1 (0.58%) |  |  |
| **Very useful** | 49 (7.46%) | 4 (7.02%) | 11 (7.75%) | 5 (8.93%) | 16 (6.99%) | 13 (7.51%) |  |  |
| **Slightly useful** | 19 (2.89%) | 2 (3.51%) | 5 (3.52%) | 5 (8.93%) | 5 (2.18%) | 2 (1.16%) |  |  |
| **Not useful at all** | 2 (0.30%) | 0 (0.00%) | 0 (0.00%) | 0 (0.00%) | 2 (0.87%) | 0 (0.00%) |  |  |
| **N/A** | 578 (87.98%) | 51 (89.47%) | 123 (86.62%) | 46 (82.14%) | 201 (87.77%) | 157 (90.75%) |  |  |
| IVR |  |  |  |  |  |  | >0.9 | 1.00 |
| **Extremely useful** | 6 (0.91%) | 0 (0.00%) | 2 (1.41%) | 0 (0.00%) | 2 (0.87%) | 2 (1.16%) |  |  |
| **Very useful** | 16 (2.44%) | 3 (5.26%) | 2 (1.41%) | 1 (1.79%) | 5 (2.18%) | 5 (2.89%) |  |  |
| **Slightly useful** | 8 (1.22%) | 0 (0.00%) | 3 (2.11%) | 1 (1.79%) | 2 (0.87%) | 2 (1.16%) |  |  |
| **Not useful at all** | 5 (0.76%) | 1 (1.75%) | 2 (1.41%) | 0 (0.00%) | 1 (0.44%) | 1 (0.58%) |  |  |
| **N/A** | 622 (94.67%) | 53 (92.98%) | 133 | 54 (96.43%) | 219 (95.63%) | 163 (94.22%) |  |  |
| Websites |  |  |  |  |  |  | 0.80 | 1.00 |
| **Extremely useful** | 9 (1.37%) | 1 (1.75%) | 1 (0.70%) | 0 (0.00%) | 4 (1.75%) | 3 (1.73%) |  |  |
| **Very useful** | 33 (5.02%) | 3 (5.26%) | 7 (4.93%) | 4 (7.14%) | 13 (5.68%) | 6 (3.47%) |  |  |
| **Slightly useful** | 22 (3.35%) | 2 (3.51%) | 8 (5.63%) | 2 (3.57%) | 6 (2.62%) | 4 (2.31%) |  |  |
| **Not useful at all** | 0 (0.00%) | 0 (0.00%) | 0 (0.00%) | 0 (0.00%) | 0 (0.00%) | 0 (0.00%) |  |  |
| **No response** | 593 (90.23%) | 51 (89.47%) | 126 (88.73%) | 50 (89.29%) | 206 (89.96%) | 160 (92.49%) |  |  |
| Mobile apps |  |  |  |  |  |  | 0.05 | 0.35 |
| **Extremely useful** | 21 (3.20%) | 1 (1.75%) | 6 (4.23%) | 0 (0.00%) | 8 (3.49%) | 6 (3.47%) |  |  |
| **Very useful** | 49 (7.46%) | 2 (3.51%) | 17 (11.97%) | 6 (10.71%) | 12 (5.24%) | 12 (6.94%) |  |  |
| **Slightly useful** | 27 (4.11%) | 4 (7.02%) | 8 (5.63%) | 3 (5.36%) | 10 (4.37%) | 2 (1.16%) |  |  |
| **Not useful at all** | 2 (0.30%) | 0 (0.00%) | 1 (0.70%) | 0 (0.00%) | 1 (0.44%) | 0 (0.00%) |  |  |
| **N/A** | 558 (84.93%) | 50 (87.72%) | 110 (77.46%) | 47 (83.93%) | 198 (86.46%) | 153 (88.44%) |  |  |

**Table S4** – **Perceived Willingness to Use Medication Adherence-Supporting Tools in the Future**

| Do you think you will start or continue recommending the following adherence tool in the next 12 months? | ALL | Geriatrician/Internist  N = 57 | General pratictioner  N = 142 | Other MD  N = 56 | Pharmacist  N = 229 | Nurse  N = 173 | p-value | q-value |
| --- | --- | --- | --- | --- | --- | --- | --- | --- |
| Patient's paper diary |  |  |  |  |  |  | <0.001 | <0.001 |
| **Definitely yes** | 125 (19,03%) | 9 (15,79%) | 36 (25,35%) | 12 (21,43%) | 45 (19,65%) | 23 (13,29%) |  |  |
| **Probably yes** | 251 (38,20%) | 19 (33,33%) | 47 (33,10%) | 22 (39,29%) | 114 (49,78%) | 49 (28,32%) |  |  |
| **Probably no** | 144 (21,92%) | 16 (28,07%) | 36 (25,35%) | 16 (28,57%) | 36 (15,72%) | 40 (23,12%) |  |  |
| **Definitely no** | 66 (10,05%) | 10 (17,54%) | 10 (7,04%) | 2 (3,57%) | 5 (2,18%) | 39 (22,54%) |  |  |
| **No response** | 71 (10,81%) | 3 (5,26%) | 13 (9,15%) | 4 (7,14%) | 29 (12,66%) | 22 (12,72%) |  |  |
| Traditional Pillbox |  |  |  |  |  |  | <0.001 | <0.001 |
| **Definitely yes** | 141 (21,46%) | 10 (17,54%) | 49 (34,51%) | 10 (17,86%) | 54 (23,58%) | 18 (10,40%) |  |  |
| **Probably yes** | 309 (47,03%) | 30 (52,63%) | 60 (42,25%) | 27 (48,21%) | 132 (57,64%) | 60 (34,68%) |  |  |
| **Probably no** | 118 (17,96%) | 8 (14,04%) | 22 (15,49%) | 17 (30,36%) | 34 (14,85%) | 37 (21,39%) |  |  |
| **Definitely no** | 83 (12,63%) | 9 (15,79%) | 10 (7,04%) | 1 (1,79%) | 7 (3,06%) | 56 (32,37%) |  |  |
| **No response** | 6 (0,91%) | 0 (0,00%) | 1 (0,70%) | 1 (1,79%) | 2 (0,87%) | 2 (1,16%) |  |  |
| Electronic pillbox |  |  |  |  |  |  | <0.001 | 0,003 |
| **Definitely yes** | 30 (4,57%) | 4 (7,02%) | 6 (4,23%) | 1 (1,79%) | 11 (4,80%) | 8 (4,62%) |  |  |
| **Probably yes** | 164 (24,96%) | 14 (24,56%) | 39 (27,46%) | 19 (33,93%) | 61 (26,64%) | 31 (17,92%) |  |  |
| **Probably no** | 213 (32,42%) | 16 (28,07%) | 42 (29,58%) | 21 (37,50%) | 91 (39,74%) | 43 (24,86%) |  |  |
| **Definitely no** | 125 (19,03%) | 11 (19,30%) | 26 (18,31%) | 7 (12,50%) | 26 (11,35%) | 55 (31,79%) |  |  |
| **No response** | 125 (19,03%) | 12 (21,05%) | 29 (20,42%) | 8 (14,29%) | 40 (17,47%) | 36 (20,81%) |  |  |
| Sms Reminder |  |  |  |  |  |  | <0.001 | 0,003 |
| **Definitely yes** | 22 (3,35%) | 0 (0,00%) | 7 (4,93%) | 0 (0,00%) | 10 (4,37%) | 5 (2,89%) |  |  |
| **Probably yes** | 169 (25,72%) | 16 (28,07%) | 35 (24,65%) | 16 (28,57%) | 73 (31,88%) | 29 (16,76%) |  |  |
| **Probably no** | 202 (30,75%) | 15 (26,32%) | 45 (31,69%) | 24 (42,86%) | 72 (31,44%) | 46 (26,59%) |  |  |
| **Definitely no** | 146 (22,22%) | 15 (26,32%) | 35 (24,65%) | 5 (8,93%) | 28 (12,23%) | 63 (36,42%) |  |  |
| **No response** | 118 (17,96%) | 11 (19,30%) | 20 (14,08%) | 11 (19,64%) | 46 (20,09%) | 30 (17,34%) |  |  |
| IVR |  |  |  |  |  |  | 0,005 | 0,035 |
| **Definitely yes** | 10 (1,52%) | 0 (0,00%) | 4 (2,82%) | 0 (0,00%) | 3 (1,31%) | 3 (1,73%) |  |  |
| **Probably yes** | 89 (13,55%) | 8 (14,04%) | 18 (12,68%) | 5 (8,93%) | 39 (17,03%) | 19 (10,98%) |  |  |
| **Probably no** | 215 (32,72%) | 15 (26,32%) | 49 (34,51%) | 23 (41,07%) | 84 (36,68%) | 44 (25,43%) |  |  |
| **Definitely no** | 176 (26,79%) | 18 (31,58%) | 42 (29,58%) | 13 (23,21%) | 39 (17,03%) | 64 (36,99%) |  |  |
| **No response** | 167 (25,42%) | 16 (28,07%) | 29 (20,42%) | 15 (26,79%) | 64 (27,95%) | 43 (24,86%) |  |  |
| Websites |  |  |  |  |  |  | 0.001 | 0.010 |
| **Definitely yes** | 25 (3,81%) | 1 (1,75%) | 6 (4,23%) | 1 (1,79%) | 12 (5,24%) | 5 (2,89%) |  |  |
| **Probably yes** | 144 (21,92%) | 11 (19,30%) | 30 (21,13%) | 13 (23,21%) | 61 (26,64%) | 29 (16,76%) |  |  |
| **Probably no** | 204 (31,05%) | 14 (24,56%) | 43 (30,28%) | 21 (37,50%) | 77 (33,62%) | 49 (28,32%) |  |  |
| **Definitely no** | 155 (23,59%) | 17 (29,82%) | 37 (26,06%) | 9 (16,07%) | 32 (13,97%) | 60 (34,68%) |  |  |
| **No response** | 129 (19,63%) | 14 (24,56%) | 26 (18,31%) | 12 (21,43%) | 47 (20,52%) | 30 (17,34%) |  |  |
| Mobile apps |  |  |  |  |  |  | 0.003 | 0.021 |
| **Definitely yes** | 36 (5,48%) | 1 (1,75%) | 11 (7,75%) | 2 (3,57%) | 13 (5,68%) | 9 (5,20%) |  |  |
| **Probably yes** | 170 (25,88%) | 13 (22,81%) | 39 (27,46%) | 15 (26,79%) | 69 (30,13%) | 34 (19,65%) |  |  |
| **Probably no** | 203 (30,90%) | 13 (22,81%) | 39 (27,46%) | 20 (35,71%) | 82 (35,81%) | 49 (28,32%) |  |  |
| **Definitely no** | 141 (21,46%) | 17 (29,82%) | 32 (22,54%) | 11 (19,64%) | 29 (12,66%) | 52 (30,06%) |  |  |
| **No response** | 107 (16,29%) | 13 (22,81%) | 21 (14,79%) | 8 (14,29%) | 36 (15,72%) | 29 (16,76%) |  |  |

**Questionnaire – Italian version**

**Indagine sugli strumenti per migliorare appropriatezza prescrittiva e aderenza alla terapia**

Gentile Collega,

L'Università di Bologna sta conducendo un'indagine in Emilia-Romagna per comprendere quanto siano diffusi e utili gli strumenti a disposizione del personale sanitario per favorire l'appropriatezza della prescrizione medica e l'aderenza al trattamento farmacologico (esempio: criteri di inappropriatezza prescrittiva nell'anziano e relative applicazioni elettroniche, diario clinico per il paziente e sistemi di monitoraggio dell'assunzione dei medicinali).

Le domande che le verranno poste sono state predisposte sulla base di una mappatura della letteratura sui principali strumenti utilizzati dal personale sanitario a livello internazionale e validate da un piccolo campione di professionisti sanitari locali.

La sua partecipazione al questionario consentirà di comprendere come migliorare lo sviluppo di tali strumenti, la loro diffusione e la formazione degli operatori sanitari per un loro utilizzo più efficiente.

Il questionario è strutturato in affermazioni sulle quali fornire un'opinione in scala strutturata (esempio, da "moltissimo" a "per niente") e domande aperte. La preghiamo di completare il questionario in tutte le sue parti e nel modo più esaustivo possibile. La partecipazione richiederà circa 10 minuti del suo tempo e le risposte fornite saranno utilizzate solo per scopi di ricerca. I risultati saranno pubblicati in forma di articolo scientifico e saranno diffusi dalle istituzioni sanitarie locali deII'EmiIia-Romagna. Sarà anche possibile ottenerli direttamente dal gruppo di ricerca dietro apposita richiesta all’indirizzo di posta elettronica riportato in fondo al messaggio. Questo studio rispetterà rigorosamente l’anonimato dei partecipanti.

Nel ringraziarla per la partecipazione, Le porgiamo Cordiali Saluti,

Elisabetta Poluzzi, Valentina Giunchi, Carlotta Lunghi, Giulia Grillini, Valentina Colonnello Dipartimento di Scienze Mediche e Chirurgiche

Alma Mater Studiorum - Università di Bologna Contatti: [elisabetta.poluzzi@unibo.it](mailto:elisabetta.poluzzi@unibo.it)

* Obbligatoria

1. Dopo aver preso visione dello scopo della ricerca e della modalità di utilizzo dei dati, le chiediamo il consenso alla partecipazione alla ricerca e all'utilizzo dei suoi dati in forma anonima *

- Ho compreso l’informativa e acconsento alla partecipazione
- Non partecipo (uscita dal modulo)

1. Genere *

- Maschio
- Femmina
- Altro/preferisco non specificarlo

1. Età *

- Meno di 40 anni
- Tra i 40 e i 60 anni
- Più di 60 anni

1. Professione *

- Medico di base
- Geriatra
- Farmacista
- Infermiere
- Altro

1. Indichi di seguito da quanti anni svolge la sua attuale professione. Se svolge la professione da meno di 1 anno, indichi "< 1" *
2. Considerando gli ultimi 12 mesi, in media, quanti pazienti vede ogni SETTIMANA? *

1. Considerando gli ultimi 12 mesi, in media, quanti pazienti di età superiore ai 65 anni, vede ogni SETTIMANA? *

# **ADERENZA AL TRATTAMENTO**

1. Per migliorare l'aderenza al trattamento farmacologico, a quanti pazienti over 65 ha consigliato negli ultimi 12 mesi l'utilizzo di: *

DIARIO CARTACEO DI AUTO-MONITORAGGIO DELLA TERAPIA?

- A nessun paziente
- A meno della metà dei pazienti
- A più della metà dei pazienti
- A tutti i pazienti
- Non Io conosco

1. Considerando la sua esperienza, quanto ritiene utile l'utilizzo del DIARIO CARTACEO DI AUTO-MONITORAGGIO DELLA TERAPIA per l'aderenza al trattamento? *

- Per niente
- Poco
- Molto
- Moltissimo

1. Quali sono, in base alla sua esperienza, le difficoltà e gli ostacoli legati all'utilizzo del DIARIO CARTACEO DI AUTO-MONITORAGGIO DELLA TERAPIA?
2. Pensa che nei prossimi 12 mesi inizierà o continuerà a suggerire l'utilizzo del DIARIO CARTACEO DI AUTO-MONITORAGGIO DELLA TERAPIA per migliorare l'aderenza del paziente? *

- Sicuramente no
- Probabilmente no
- Probabilmente sì
- Sicuramente sì

1. Per migliorare l'aderenza al trattamento farmacologico, a quanti pazienti over 65 ha consigliato negli ultimi 12 mesi l'utilizzo di: *

PORTAPILLOLE TRADIZIONALE CON SUDDIVISIONE GIORNALIERA?

- A nessun paziente
- A meno della metà dei pazienti
- A più della metà dei pazienti
- A tutti i pazienti
- Non Io conosco

1. Considerando la sua esperienza, quanto ritiene utile l'utilizzo del PORTAPILLOLE TRADIZIONALE CON SUDDIVISIONE GIORNALIERA per l'aderenza al trattamento? *

- Per niente
- Poco
- Molto
- Moltissimo

1. Quali sono, in base alla sua esperienza, le difficoltà e gli ostacoli legati all'utilizzo del PORTAPILLOLE TRADIZIONALE CON SUDDIVISIONE GIORNALIERA?
2. Pensa che nei prossimi 12 mesi inizierà o continuerà a suggerire l'utilizzo del PORTAPILLOLE TRADIZIONALE CON SUDDIVISIONE GIORNALIERA per migliorare l'aderenza del paziente? *

- Sicuramente no
- Probabilmente no
- Probabilmente sì
- Sicuramente sì

1. Per migliorare l'aderenza al trattamento farmacologico, a quanti pazienti over 65 ha consigliato negli ultimi 12 mesi l'utilizzo di: *

PORTAPILLOLE ELETTRONICO (CON PROMEMORIA E/O REGISTRAZIONE DELL'APERTURA)?

- A nessun paziente
- A meno della metà dei pazienti
- A più della metà dei pazienti
- A tutti i pazienti
- Non Io conosco

1. Considerando la sua esperienza, quanto ritiene utile l'utilizzo del PORTAPILLOLE ELETTRONICO (CON PROMEMORIA E/O REGISTRAZIONE DELL'APERTURA) per l'aderenza al trattamento? *

- Per niente
- Poco
- Molto
- Moltissimo

1. Quali sono, in base alla sua esperienza, le difficoltà e gli ostacoli legati all'utilizzo del PORTAPILLOLE ELETTRONICO (CON PROMEMORIA E/O REGISTRAZIONE DELL'APERTURA)?
2. Pensa che nei prossimi 12 mesi inizierà o continuerà a suggerire l'utilizzo del PORTAPILLOLE ELETTRONICO (CON PROMEMORIA E/O REGISTRAZIONE DELL'APERTURA) per migliorare l'aderenza del paziente?

- Sicuramente no
- Probabilmente no
- Probabilmente sì
- Sicuramente sì

1. Per migliorare l'aderenza al trattamento farmacologico, a quanti pazienti over 65 ha consigliato negli ultimi 12 mesi l'utilizzo di: *

SERVIZI DI MESSAGGISTICA (SMS o MMS) MHEALTH?

- A nessun paziente
- A meno della metà dei pazienti
- A più della metà dei pazienti
- A tutti i pazienti
- Non Io conosco

1. Considerando la sua esperienza, quanto ritiene utile l'utilizzo dei SERVIZI DI MESSAGGISTICA (SMS o MMS) MHEALTH per l'aderenza al trattamento? *

- Per niente
- Poco
- Molto
- Moltissimo

1. Quali sono, in base alla sua esperienza, le difficoltà e gli ostacoli legati all'utilizzo dei SERVIZI DI MESSAGGISTICA (SMS o MMS) MHEALTH?
2. Pensa che nei prossimi 12 mesi inizierà o continuerà a suggerire l'utilizzo di SERVIZI DI MESSAGGISTICA (SMS o MMS) MHEALTH per migliorare l'aderenza del paziente? *

- Sicuramente no
- Probabilmente no
- Probabilmente sì
- Sicuramente sì

1. Per migliorare l'aderenza al trattamento farmacologico, a quanti pazienti over 65 ha consigliato negli ultimi 12 mesi l'utilizzo di: *

SISTEMI DI RISPOSTA VOCALE INTERATTIVA (IVR)?

- A nessun paziente
- A meno della metà dei pazienti
- A più della metà dei pazienti
- A tutti i pazienti
- Non Io conosco

1. Considerando la sua esperienza, quanto ritiene utile l'utilizzo dei SISTEMI DI RISPOSTA VOCALE INTERATTIVA (IVR) per l'aderenza al trattamento? *

- Per niente
- Poco
- Molto
- Moltissimo

1. Quali sono, in base alla sua esperienza, le difficoltà e gli ostacoli legati all'utilizzo dei SISTEMI DI RISPOSTA VOCALE INTERATTIVA (IVR)?
2. Pensa che nei prossimi 12 mesi inizierà o continuerà a suggerire l'utilizzo di SISTEMI DI RISPOSTA VOCALE INTERATTIVA (IVR) per migliorare l'aderenza del paziente? *

- Sicuramente no
- Probabilmente no
- Probabilmente sì
- Sicuramente sì

1. Per migliorare l'aderenza al trattamento farmacologico, a quanti pazienti over 65 ha consigliato negli ultimi 12 mesi l'utilizzo di: *

SITI WEB EDUCATIVI E DI MONITORAGGIO TRAMITE FEEDBACK DA PARTE DI OPERATORI SANITARI?

- A nessun paziente
- A meno della metà dei pazienti
- A più della metà dei pazienti
- A tutti i pazienti
- Non Io conosco

1. Considerando la sua esperienza, quanto ritiene utile l'utilizzo dei SITI WEB EDUCATIVI E DI MONITORAGGIO TRAMITE FEEDBACK DA PARTE DI OPERATORI SANITARI per l'aderenza al trattamento? *

- Per niente
- Poco
- Molto
- Moltissimo

1. Quali sono, in base alla sua esperienza, le difficoltà e gli ostacoli legati all'utilizzo dei SITI WEB EDUCATIVI E DI MONITORAGGIO TRAMITE FEEDBACK DA PARTE DI OPERATORI SANITARI?
2. Pensa che nei prossimi 12 mesi inizierà o continuerà a suggerire l'utilizzo di SITI WEB EDUCATIVI E DI MONITORAGGIO TRAMITE FEEDBACK DA PARTE DI OPERATORI SANITARI per migliorare l'aderenza del paziente? *

- Sicuramente no
- Probabilmente no
- Probabilmente sì
- Sicuramente sì

1. Per migliorare l'aderenza al trattamento farmacologico, a quanti pazienti over 65 ha consigliato negli ultimi 12 mesi l'utilizzo di: *

APP EDUCATIVE CON FUNZIONE DI PROMEMORIA E/O CHE CONSENTONO DI COMUNICARE CON IL MEDICO CURANTE 0 ALTRO PERSONALE SANITARIO?

- A nessun paziente
- A meno della metà dei pazienti
- A più della metà dei pazienti
- A tutti i pazienti
- Non Io conosco

1. Considerando la sua esperienza, quanto ritiene utile l'utilizzo di APP EDUCATIVE CON FUNZIONE DI PROMEMORIA E/O CHE CONSENTONO DI COMUNICARE CON IL MEDICO CURANTE 0 ALTRO PERSONALE SANITARIO per l'aderenza al trattamento? *

- Per niente
- Poco
- Molto
- Moltissimo

1. Quali sono, in base alla sua esperienza, le difficoltà e gli ostacoli legati all'utilizzo di APP EDUCATIVE CON FUNZIONE DI PROMEMORIA E/O CHE CONSENTONO DI COMUNICARE CON IL MEDICO CURANTE 0 ALTRO PERSONALE SANITARIO?
2. Pensa che nei prossimi 12 mesi inizierà o continuerà a suggerire l'utilizzo di APP EDUCATIVE CON FUNZIONE DI PROMEMORIA E/O CHE CONSENTONO DI COMUNICARE CON IL MEDICO CURANTE 0 ALTRO PERSONALE SANITARIO per migliorare l'aderenza del paziente? *

- Sicuramente no
- Probabilmente no
- Probabilmente sì
- Sicuramente sì

# **APROPRIATEZZA PRESCRITTIVA**

1. Per favorire l'APPROPRIATEZZA dei trattamenti farmacologici neII'anziano è possibile far riferimento ai criteri BEERS, una lista di farmaci suddivisa in tre sezioni:

- farmaci da evitare
- farmaci potenzialmente inappropriati in determinate condizioni
- farmaci da utilizzare con cautela

Per maggiori informazioni: By the 2019 American Geriatrics Society Beersb criteria Update Expert Panel. American Geriatrics Society 2019 Updated AGS Beers Criteria for Potentially Inappropriate Medication Use in Older

Adults. Journal of the American Geriatrics Society vol. 67,4 (2019): 674-694. doi:10.1 1 1 1/jgs.15767

Negli ultimi 1 2 mesi, si è avvalso/a dei criteri di BEERS

- Per nessun paziente
- Per meno della metà dei pazienti
- Per più della metà dei pazienti
- Per tutti i pazienti
- Non li conosco

1. Per favorire l’APPROPRIATEZZA nella prescrizione è possibile seguire i criteri START/STOPP.

I criteri STOPP (Screening Tool of Older Person’s Prescriptions) identificano farmaci da evitare neII‘anziano perché non adatti per posologia o durata della terapia.

I criteri START (Screening Tool to Alert Doctor to Right Treatment) identificano farmaci con chiaro beneficio in presenza di una precisa diagnosi neII'anziano.

Per maggiori informazioni: Diaz Planelles I, et al. Prevalence of Potentially Inappropriate Prescriptions According to the New STOPP/START Criteria in Nursing Homes: A Systematic Review. Healthcare (Basel). 2023;1 1(3):422. Published 2023 Feb 1. doi:10.3390/heaIthcare1 1030422 Negli ultimi 1 2 mesi, si è avvalso/a dei criteri di START/STOPP

- Per nessun paziente
- Per meno della metà dei pazienti
- Per più della metà dei pazienti
- Per tutti i pazienti
- Non li conosco

1. Per favorire l’APPROPRIATEZZA PRESCRITTIVA si possono seguire linee guida e protocolli di pratica clinica come MULTIPAP (Improving Healthcare in Multimorbidity and Polypharmacy in Primary Care - Principi di Ariadne).

Per maggiori informazioni: Del Cura-Gonzàlez I, et al. How to Improve Healthcare for Patients with Multimorbidity and Polypharmacy in Primary Care: A Pragmatic Cluster-Randomized Clinical Trial of the MULTIPAP Intervention. 7 Pers Med. 2022;12(5):752. Published 2022 May 6. doi:10.3390/jpm12050752

Negli ultimi 1 2 mesi ha seguito questi protocolli *

- Per nessun paziente
- Per meno della metà dei pazienti
- Per più della metà dei pazienti
- Per tutti i pazienti
- Non li conosco

1. Per favorire l’APPROPRIATEZZA PRESCRITTIVA sono utili software di supporto alla prescrizione, ossia programmi informatici che consentono la ricettazione elettronica a partire dalla cartella clinica elettronica del paziente e che possono includere raccomandazioni sulle terapie farmacologiche per Io specifico paziente (tali raccomandazioni si basano su algoritmi che derivano da linee guida e criteri di appropriatezza/inappropriatezza, si veda anche domande 36-38).

Negli ultimi 1 2 mesi si è avvalso/a di tali strumenti *

- Per nessun paziente
- Per meno della metà dei pazienti
- Per più della metà dei pazienti
- Per tutti i pazienti
- Non li conosco

1. Per favorire l’APPROPRIATEZZA PRESCRITTIVA possono essere utilizzati numerosi database contenenti informazioni evidence-based su farmaci, Ioro profilo di sicurezza, rischio di interazioni, eventuali monitoraggi utili.

Negli ultimi 1 2 mesi si è avvalso/a di *

|  | | Per meno | Per più |  | |
| --- | --- | --- | --- | --- | --- |
|  |  | della metà | della metà |  |  |
|  | Per nessun | dei | dei | Per tutti i | Non Io |
| Micromedex | paziente  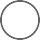 | pazienti  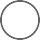 | pazienti  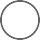 | pazienti  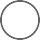 | conosco  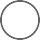 |
| UpToDate | 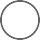 | 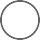 | 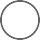 | 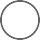 | 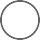 |
|  |  |  |  |  |  |

1. Per favorire l’APPROPRIATEZZA PRESCRITTIVA può essere utilizzato INTERCheck, un sistema di supporto alle prescrizioni che ha l'obiettivo di bilanciare rischi e benefici di una terapia fornendo un un punteggio di rischio di potenziali interazioni farmacologiche.

Per maggiori informazioni: <https://intercheckweb.marionegri.it/>

Negli ultimi 12 mesi si è avvalso/a di tale strumento

- Per nessun paziente
- Per meno della metà dei pazienti
- Per più della metà dei pazienti
- Per tutti i pazienti
- Non Io conosco

1. Negli ultimi 12 mesi, di quali ulteriori strumenti si è avvalso/a?
2. Ci sono stati specifici ostacoli all'utilizzo degli strumenti per favorire l'appropriatezza che ha utilizzato? (le chiediamo di definire gli ostacoli specifici per singolo strumento utilizzato)
3. Cosa potrebbe essere utile per supportarla nell'attività di monitoraggio deII'APPROPRIATEZZA PRESCRITTIVA in futuro?
4. Frequenterebbe un corso o seguirebbe un tutorial di circa 1 ora sugli strumenti a disposizione?*

|  | Assolutamente no | Probabilmente no | Probabilmente si | Assolutamente si |
| --- | --- | --- | --- | --- |
| Per l’aderenza  al trattamento | 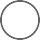 | 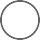 | 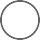 | 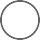 |
| Per l’appropriatezza  prescrittiva | 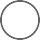 | 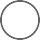 | 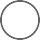 | 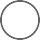 |

1. La ringraziamo per la partecipazione. Se Io desidera, può utilizzare questo spazio per condividere un'opinione o un commento sul questionario o su aspetti che non sono stati affrontati nella presente indagine e che ritiene utili per l'integrazione alle risposte già fornite.

Gentile collega, la sua esperienza sarebbe un contributo prezioso per la nostra ricerca

Siamo consapevoli che la sua professione richiede un impegno costante.

La ringraziamo in ogni caso per aver preso in considerazione il nostro questionario e rispettiamo la sua scelta di non partecipare.

Grazie per aver dedicato il suo tempo a questa ricerca.

Desideriamo esprimerle la nostra più sincera gratitudine per aver collaborato allo studio.


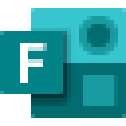
Questo contenuto non è stato creato né approvato da Microsoft. I dati che invii verranno recapitati al proprietario del modulo. Microsoft Forms

**Questionnaire – English translated version**

**Survey on Tools to Improve Prescriptive Appropriateness and Adherence to Therapy**

Dear Colleague,

The University of Bologna is conducting a survey in the Emilia-Romagna region to assess the prevalence and effectiveness of tools available to healthcare professionals for promoting appropriate medical prescriptions and adherence to pharmacological treatments (e.g., criteria for prescribing appropriateness in older adults and related electronic applications, clinical diaries for patients, and systems for monitoring medication intake).

The survey questions were developed based on a literature review of primary tools used by healthcare professionals worldwide and validated with a small sample of local healthcare professionals.

Your participation in this questionnaire is invaluable in helping us understand how to improve the development and dissemination of these tools and enhance the training of healthcare professionals to optimize their use.

The questionnaire consists of statements rated on a structured scale (e.g., from “very much” to “not at all”) and open-ended questions. Please complete the questionnaire in full and provide responses as comprehensively as possible. Completing the survey will take approximately 10 minutes of your time, and all responses will be used solely for research purposes.

The results of this study will be published as a scientific article and disseminated by local health institutions in Emilia-Romagna. Additionally, they can be obtained directly by contacting the research team at the email address provided below. This study fully guarantees the anonymity of all participants.

Thank you for participating.

Best regards,

Elisabetta Poluzzi, Valentina Giunchi, Carlotta Lunghi, Giulia Grillini, Valentina Colonnello Department of Medical and Surgical Sciences

Alma Mater Studiorum – University of Bologna

Contact: [elisabetta.poluzzi@unibo.it](mailto:elisabetta.poluzzi@unibo.it)

* Mandatory

1. After reviewing the purpose of the research and the data usage method, we ask for your consent to participate in the research and to use your data anonymously *

- I have read the information and consent to participate
- I do not consent to participate (exit the form)

1. Gender *

- Male
- Female
- Other/Prefer not to specify

1. Age *

- Under 40 years
- Between 40 and 60 years
- Over 60 years

1. Profession *

- General practitioner
- Geriatrician
- Pharmacist
- Nurse
- Other

1. Indicate below how many years you have been in your current profession. If you have been in the profession for less than 1 year, please indicate "< 1" *
2. Considering the last 12 months, on average, how many patients do you see per week? *
3. Considering the last 12 months, on average, how many patients over 65 years of age do you see per week?*

# **ADHERENCE TO TREATMENT**

1. To improve adherence to pharmacological treatment, how many patients over 65 have you recommended in the last few months to use the following tools?

PAPER DIARY FOR SELF-MONITORING OF THERAPY *

- None
- Less than half of the patients
- More than half of the patients
- All patients
- Not known

1. Based on your experience, how useful do you consider the use of the

PAPER DIARY FOR SELF-MONITORING OF THERAPY in improving treatment

adherence? *

- Not at all
- Slightly
- Very
- Extremely

1. Based on your experience, what are the difficulties and obstacles related to the use of the PAPER DIARY FOR SELF-MONITORING OF THERAPY?

11. Do you think you will start or continue recommending the use of PAPER DIARY FOR SELF-MONITORING OF THERAPY in the next 12 months to improve treatment adherence? *

- Definitely not
- Probably not
- Probably yes
- Definitely yes

1 2. To improve adherence to pharmacological treatment, how many patients over 65 have you recommended in the last few months to use the following instrument:

TRADITIONAL PILLBOX WITH DAILY DIVISION? *

- None
- Less than half of the patients
- More than half of the patients
- All patients
- Not known

1. Based on your experience, how useful do you consider the use of the TRADITIONAL PILLBOX WITH DAILY DIVISION to improve treatment adherence? *

- Not at all
- Slightly
- Very
- Extremely

1. Based on your experience, what are the difficulties and obstacles related to the use of the TRADITIONAL PILLBOX WITH DAILY DIVISION?
2. Do you think you will start or continue recommending the use of TRADITIONAL PILLBOX WITH DAILY DIVISION in the next 12 months to improve patient adherence? *

- Definitely not
- Probably no t
- Probably yes
- Definitely yes

1. To improve adherence to pharmacological treatment, how many patients over 65 have you recommended in the last few months to use the following instrument:

ELECTRONIC PILLBOX (WITH REMINDER AND/OR RECORDING OF OPENING)? *

- None
- Less than half of the patients
- More than half of the patients
- All patients
- Not known

1. Based on your experience, how useful do you consider the use of the ELECTRONIC PILLBOX (WITH REMINDER AND/OR RECORDING OF OPENING) to improve treatment adherence? *

- Not at all
- Slightly
- Very
- Extremely

1. Based on your experience, what are the difficulties and obstacles related to the use of the ELECTRONIC PILLBOX (WITH REMINDER AND/OR RECORDING OF OPENING)?
2. Do you think you will start or continue recommending the use of ELECTRONIC PILLBOX (WITH REMINDER AND/OR RECORDING OF OPENING) in the next 12 months to improve patient adherence? *

- Definitely not
- Probably not
- Probably yes
- Definitely yes

1. To improve adherence to pharmacological treatment, how many patients over 65 have you recommended in the last few months to use the following instrument:

MHEALTH MESSAGE SERVICES (SMS or MMS)? *

- None
- Less than half of the patients
- More than half of the patients
- All patients
- Not known

1. Based on your experience, how useful do you consider the use of the MHEALTH MESSAGE SERVICES (SMS or MMS) to improve treatment adherence? *

- Not at all
- Slightly
- Very
- Extremely

1. Based on your experience, what are the difficulties and obstacles related to the use of the MHEALTH MESSAGE SERVICES (SMS or MMS)?
2. Do you think you will start or continue recommending the use of MHEALTH MESSAGE SERVICES (SMS or MMS) in the next 12 months to improve patient adherence? *

- Definitely not
- Probably not
- Probably yes
- Definitely yes

1. To improve adherence to pharmacological treatment, how many patients over 65 have you recommended in the last few months to use the following instrument:

INTERACTIVE VOICE RESPONSE SYSTEMS (IVR)? *

- None
- Less than half of the patients
- More than half of the patients
- All patients
- Not known

1. Based on your experience, how useful do you consider the use of the INTERACTIVE VOICE RESPONSE SYSTEMS (IVR) to improve treatment adherence? *

- Not at all
- Slightly
- Very
- Extremely

1. Based on your experience, what are the difficulties and obstacles related to the use of the INTERACTIVE VOICE RESPONSE SYSTEMS (IVR)?
2. Do you think you will start or continue recommending the use of INTERACTIVE VOICE RESPONSE SYSTEMS (IVR) in the next 12 months to improve patient adherence? *

- Definitely not
- Probably not
- Probably yes
- Definitely yes

1. To improve adherence to pharmacological treatment, how many patients over 65 have you recommended in the last few months to use the following instrument:

EDUCATIONAL AND MONITORING WEBSITES THROUGH FEEDBACK FROM HEALTH PROFESSIONALS? *

- None
- Less than half of the patients
- More than half of the patients
- All patients
- Not known

1. Based on your experience, how useful do you consider the use of the EDUCATIONAL AND MONITORING WEBSITES THROUGH FEEDBACK FROM HEALTH PROFESSIONALS to improve treatment adherence? *

- Not at all
- Slightly
- Very much
- Extremely

1. Based on your experience, what are the difficulties and obstacles related to the use of the EDUCATIONAL AND MONITORING WEBSITES THROUGH FEEDBACK FROM HEALTH PROFESSIONALS?
2. Do you think you will start or continue recommending the use of EDUCATIONAL AND MONITORING WEBSITES THROUGH FEEDBACK FROM HEALTH PROFESSIONALS in the next 12 months to improve patient adherence? *

- Definitely not
- Probably not
- Probably yes
- Definitely yes

1. To improve adherence to pharmacological treatment, how many patients over 65 have you recommended in the last few months to use the following instrument:

EDUCATIONAL APPS WITH A REMINDER FUNCTION AND/OR COMMUNICATION WITH THE ATTENDING PHYSICIAN OR OTHER HEALTH PERSONNEL? *

- None
- Less than half of the patients
- More than half of the patients
- All patients
- Not known

1. Based on your experience, how useful do you consider the use of the EDUCATIONAL APPS WITH A REMINDER FUNCTION AND/OR COMMUNICATION WITH THE ATTENDING PHYSICIAN OR OTHER HEALTH PERSONNEL to improve treatment adherence? *

- Not at all
- Slightly
- Very
- Extremely

1. Based on your experience, what are the difficulties and obstacles related to the use of the EDUCATIONAL APPS WITH A REMINDER FUNCTION AND/OR COMMUNICATION WITH THE ATTENDING PHYSICIAN OR OTHER HEALTH PERSONNEL?
2. Do you think you will start or continue recommending the use of EDUCATIONAL APPS WITH A REMINDER FUNCTION AND/OR COMMUNICATION WITH THE ATTENDING PHYSICIAN OR OTHER HEALTH PERSONNEL in the next 12 months to improve patient adherence? *

- Definitely not
- Probably not
- Probably yes
- Definitely yes

**Prescriptive Appropriateness**

1. To promote the PRESCRIBING APPROPRIATENESS of pharmacological treatments in older adults, the BEERS CRITERIA can be referenced. This is a list of medications divided into three sections:
   -Medications to avoid
   -Medications potentially inappropriate in certain conditions
   -Medications to use with caution

For more information:

By the 2019 American Geriatrics Society Beers Criteria Update Expert Panel. American Geriatrics Society 2019 Updated AGS Beers Criteria for Potentially Inappropriate Medication Use in Older Adults. *Journal of the American Geriatrics Society* vol. 67,4 (2019): 674-694. doi:10.1 1 1 1/jgs.15767

In the last 12 months, have you used the BEERS CRITERIA? *

- For no patient
- For less than half of the patients
- For more than half of the patients
- For all patients
- I am not familiar with them

1. To promote PRESCRIBING APPROPRIATENESS in prescribing, the START/STOPP CRITERIA can be followed.
   The STOPP CRITERIA (Screening Tool of Older Person’s Prescriptions) identify medications to avoid in older adults due to inappropriate dosage or duration of therapy.
   The START criteria (Screening Tool to Alert Doctor to Right Treatment) identify medications with clear benefits for older adults with specific diagnoses.

For more information:

Diaz Planelles I, et al. Prevalence of Potentially Inappropriate Prescriptions According to the New STOPP/START Criteria in Nursing Homes: A Systematic Review. *Healthcare (Basel).* 2023;1 1(3):422. Published 2023 Feb 1. doi:10.3390/heaIthcare1 1030422

In the last 12 months, have you used the STOPP/START CRITERIA? *

- For no patient
- For less than half of the patients
- For more than half of the patients
- For all patients
- I am not familiar with them

## To promote PRESCRIBING APPROPRIATENESS, clinical practice guidelines and protocols such as MULTIPAP (Improving Healthcare in Multimorbidity and Polypharmacy in Primary Care - Ariadne Principles) can be followed.

For more information:

Del Cura-Gonzàlez I, et al. How to Improve Healthcare for Patients with Multimorbidity and Polypharmacy in Primary Care: A Pragmatic Cluster-Randomized Clinical Trial of the MULTIPAP Intervention. 7 *Pers Med.* 2022;12(5):752. Published 2022 May 6. doi:10.3390/jpm12050752

In the last 12 months, have you used MULTIPAP? *

- For no patient
- For less than half of the patients
- For more than half of the patients
- For all patients
- I am not familiar with them

1. To promote PRESCRIBING APPROPRIATENESS, prescribing SUPPORT SOFTWARE can be useful. These are computer programs that allow electronic prescriptions based on the patient's electronic health record and may include recommendations for pharmacological therapies tailored to the specific patient (such recommendations are based on algorithms derived from guidelines and appropriateness/inappropriateness criteria, see also questions 36-38).

In the last 12 months, have you used SUPPORT SOFTWARE? *

- For no patient
- For less than half of the patients
- For more than half of the patients
- For all patients
- I am not familiar with them

1. To promote APPROPRIATE PRESCRIBING, several DATABASES containing evidence-based information about medications, their safety profiles, potential interactions, and relevant monitoring requirements can be utilized.

In the last 12 months, which of the following DATABASES have you used?

|  | |  |  |  | |
| --- | --- | --- | --- | --- | --- |
|  |  |  |  |  |  |
|  | For no patient | For less than half | For more than half | For all patient | Not known |
| Micromedex | 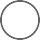 | 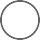 | 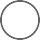 | 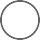 | 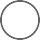 |
| UpToDate | 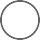 | 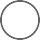 | 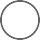 | 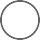 | 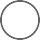 |
|  |  |  |  |  |  |

1. To promote PRESCRIBING APPROPRIATENESS, INTERCheck WEB can be useful.

INTERCheck is a prescribing support system that helps balance the risks and benefits of a therapy by providing a risk score for potential drug interactions.

For more information: https://intercheckweb.marionegri.it/

In the last 12 months, have you used SUPPORT SOFTWARE? *

- For no patient
- For less than half of the patients
- For more than half of the patients
- For all patients
- I am not familiar with them

1. What other prescribing tools have you used in the last 12 months?
2. Have you encountered specific challenges or obstacles when using the tools to promote appropriateness? (Please specify the challenges for each tool used)
3. What support would help you monitor APPROPRIATE PRESCRIBING in the future?

Would you attend a course or follow a tutorial (approximately 1 hour) on the tools available? *

|  | Definitely not | Probably not | Probably yes | Definitely yes |
| --- | --- | --- | --- | --- |
| For adherence | 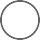 | 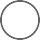 | 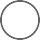 | 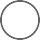 |
| For appropriateness | 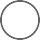 | 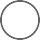 | 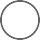 | 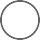 |

1. Thank you for your participation. If you wish, you can use this space to share your opinion or comment on the questionnaire or on aspects not addressed in this survey that you consider useful to complement the responses provided.

Dear colleague,

Your experience is a valuable contribution to our research. We are aware that your profession demands constant dedication.

We thank you for considering our questionnaire and respect your choice not to participate.

Thank you for taking the time to contribute to this research.

We wish to express our sincere gratitude for your collaboration in this study.

This content was neither created nor approved by Microsoft. The data you submit will be delivered to the form owner.


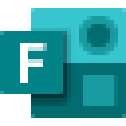
Microsoft Forms
